# A cAMP/CRP-controlled mechanism for the incorporation of extracellular ADP-glucose in *Escherichia coli* involving NupC and NupG nucleoside transporters

**DOI:** 10.1038/s41598-018-33647-w

**Published:** 2018-10-19

**Authors:** Goizeder Almagro, Alejandro M. Viale, Manuel Montero, Francisco José Muñoz, Edurne Baroja-Fernández, Hirotada Mori, Javier Pozueta-Romero

**Affiliations:** 1 Instituto de Agrobiotecnología (CSIC, UPNA, Gobierno de Navarra), Iruñako etorbidea 123, 31192 Mutiloa, Nafarroa Spain; 2 Instituto de Biología Molecular y Celular de Rosario (IBR, CONICET), Departamento de Microbiología, Facultad de Ciencias Bioquímicas y Farmacéuticas, Universidad Nacional de Rosario, Suipacha 521, 2000 Rosario, Argentina; 3 Data Science Center, Division of Biological Science, Nara Institute of Science and Technology, Ikoma, Nara 630-0101 Japan

## Abstract

ADP-glucose is the precursor of glycogen biosynthesis in bacteria, and a compound abundant in the starchy plant organs ingested by many mammals. Here we show that the enteric species *Escherichia coli* is capable of scavenging exogenous ADP-glucose for use as a glycosyl donor in glycogen biosynthesis and feed the adenine nucleotide pool. To unravel the molecular mechanisms involved in this process, we screened the *E*. *coli* single-gene deletion mutants of the Keio collection for glycogen content in ADP-glucose-containing culture medium. In comparison to wild-type (WT) cells, individual *∆nupC* and *∆nupG* mutants lacking the cAMP/CRP responsive inner-membrane nucleoside transporters NupC and NupG displayed reduced glycogen contents and slow ADP-glucose incorporation. In concordance, *∆cya* and *∆crp* mutants accumulated low levels of glycogen and slowly incorporated ADP-glucose. Two-thirds of the glycogen-excess mutants identified during screening lacked functions that underlie envelope biogenesis and integrity, including the RpoE specific RseA anti-sigma factor. These mutants exhibited higher ADP-glucose uptake than WT cells. The incorporation of either *∆crp*, *∆nupG* or *∆nupC* null alleles sharply reduced the ADP-glucose incorporation and glycogen content initially witnessed in *∆rseA* cells. Overall, the data showed that *E*. *coli* incorporates extracellular ADP-glucose through a cAMP/CRP-regulated process involving the NupC and NupG nucleoside transporters that is facilitated under envelope stress conditions.

## Introduction

*Escherichia coli* is the predominant facultative anaerobe of the commensal microbiota inhabiting the mammalian intestine, and arguably the best understood of all model bacterial organisms^1,2^. This bacterium has evolved dedicated systems for obtaining carbon and energy sources from the external environment, with the phosphotransferase system (PTS) representing the best characterized system^3,4^. The PTS catalyzes the transport and phosphorylation of several carbohydrates whose levels reflect nutrient availability and cellular energy conditions. This information is transduced through different mechanisms and culminates in the phenomenon of carbon catabolite repression (CCR), defined as the inhibition of gene expression and/or protein activity due to the presence of a rapidly metabolizable primary carbon source (frequently glucose) in the growth medium^5^. CCR involves a membrane-bound adenylate cyclase, the product of the *cya* gene, its product cyclic AMP (cAMP), which acts as a nutrient availability and energy sufficiency sensor, and the cAMP receptor protein (CRP), which acts as a transcription activator. The cAMP/CRP system plays a key role in regulating the expression of many regulons and operons that encode enzymes and transporters involved in the catabolism of different nutrient sources^2,5–7^.

Many bacterial species, including *E*. *coli*, accumulate glycogen when carbon sources are in excess but the availability of other nutrients is limited^8^. This polyglucan is synthesized by glycogen synthase (GlgA), with ADP-glucose (ADPG) produced by ADPG pyrophosphorylase (GlgC) serving as the glucosyl moiety donor, yet the exact role of glycogen in bacterial physiology is still not well defined^8^. In members of the *Enterobacteriaceae* family such as *E*. *coli*, genes involved in glycogen metabolism are organized in a single *glgBXCAP* operon^9,10^, the expression of which is tightly regulated by a complex assemblage of factors that are adjusted to the nutritional status of the cell^9,11,12^. In addition to a role in bacterial glycogen production, ADPG also acts as the glycosyl donor molecule in the reaction of starch synthesis in plants, with the content of this nucleotide-sugar in starchy organs reported to be as high as 600 nmol per gram of dry weight^13,14^. Although the diets of many mammals, including human beings, can include high proportions of ADPG-enriched plant starchy organs, the possible occurrence of systems enabling the incorporation and utilization of extracellular ADPG in enteric bacterial species has not yet been explored.

Here we show that *E*. *coli* is capable of directly incorporating ADPG from the extracellular medium. To identify the ADPG transport machinery and elucidate their regulatory properties, we carried out a genome-wide screening of the genes affecting ADPG incorporation using a systematic and comprehensive gene-disrupted *E*. *coli* mutant collection (the Keio collection^15^). We found that ADPG incorporation into *E*. *coli* cells is a cAMP/CRP-regulated process involving the NupC and NupG inner membrane transporters that is facilitated under conditions of extracytoplasmic stress. This finding extends our knowledge of the diverse mechanisms underlying physiological modulation in *E*. *coli*, and sheds light on the adaptive traits evolved by this organism to survive the conditions prevailing on the harsh, highly competitive environment of the intestine. This is the first report showing the capacity of a bacterial species of directly taking up ADPG from the external medium, also identifying the responsible mechanisms and regulatory component of this process.

## Results

### *E*. *coli* can directly incorporate extracellular ADPG

**Figure 1:**
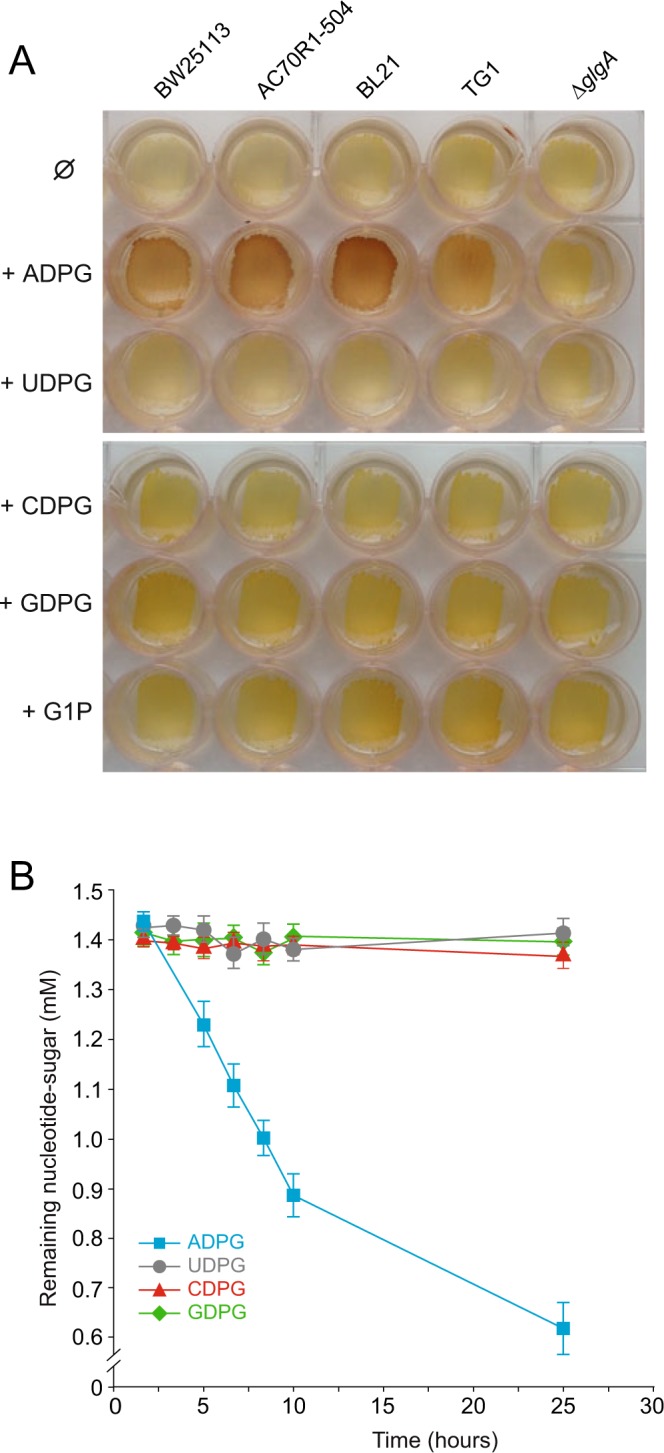
*E*. *coli* can directly incorporate extracellular ADPG. (**A**) Glycogen iodine staining of different *E*. *coli* strains (*i*.*e*. BW25113, TG1, BL21, AC70R1-504 and Δ*glgA*) cultured in KM with or without ADPG, UDPG, CDPG, GDPG or G1P supplementation, 1.5 mM each. (**B**) Time-course analysis of nucleotide-sugar consumption by BW25113 (WT) cells cultured in liquid KM supplemented with ADPG, UDPG, CDPG or GDPG (1.5 mM each). Values represent means ± SE obtained from four independent experiments with 3 replicates for each experiment. Growth curves are shown in Supplementary Fig. S3A. Time-course analyses of ADPG consumption by BW25113 cells in four independent biological replicates are shown in Supplementary Fig. S1.

As a first step to exploring the possibility of mechanism(s) that enable the direct incorporation of ADPG in *E*. *coli*, we analyzed the glycogen content in cells belonging to different *E*. *coli* lineages, including K-12 (strains BW25113, TG1) and B (strain BL21), cultured in solid Kornberg medium (KM) with or without 1.5 mM ADPG supplementation using the iodine staining technique^12^. We also analyzed the glycogen content in *E*. *coli* cells cultured in solid KM with or without other nucleotide-sugars (*i*.*e*. UDP-glucose, CDP-glucose and GDP-glucose). GlgA null *∆glgA* cells that are not capable of producing glycogen from ADPG served as negative control. As shown in Fig. 1A, we found that wild-type (WT) cells cultured in solid KM supplemented with ADPG (KM-ADPG) stained darker than WT cells cultured in ADPG-free KM independent of the *E*. *coli* strain used. On the contrary, no substantial glycogen accumulation was observed in the same cells grown in KM supplemented with UDP-glucose (UDPG), CDP-glucose (CDPG) or GDP-glucose (GDPG) or, as expected, in *∆glgA* cells cultured in KM-ADPG.

*E*. *coli* strains are equipped with periplasmic hydrolases that potentially enable the conversion of extracellular ADPG into G1P and/or glucose, which, once incorporated into the cell by means of hexose-phosphate or glucose transporters, could be channeled towards glycogen production by the stepwise reactions of Pgm, GlgC and GlgA^8^. To test this possibility we conducted time-course analyses of ADPG consumption by measuring ADPG remaining in the culture medium of liquid KM-ADPG grown WT cells, and the appearance of ADPG breakdown products (*i*.*e*. AMP, ADP, glucose and G1P) in the cultures. Furthermore, we carried out time-course analyses of CDPG, UDPG and GDPG consumption. As shown in Fig. 1B and Supplementary Fig. S1, the ADPG remaining in the liquid culture medium gradually declined with time, demonstrating steady ADPG consumption. In contrast, CDPG, UDPG and GDPG levels remained unaltered (Fig. 1B). G1P, glucose, AMP or ADP could not be detected in the liquid culture medium even after prolonged culturing of the cells in KM-ADPG (not shown). We also used iodine staining to study glycogen content in *E*. *coli* cells cultured in solid KM with G1P supplementation. Moreover, we analyzed the glycogen content in KM-ADPG grown GlgC null AC70R1-504 mutant cells, which are unable to produce ADPG from G1P as a consequence of an inactivating single point mutation in *glgC*^16^. As shown in Fig. 1A, *E*. *coli* cells cultured in solid KM supplemented with G1P displayed a yellow iodine staining phenotype representing low glycogen content. Moreover, AC70R1-504 cells cultured in solid KM-ADPG displayed a WT, brown iodine staining phenotype. Taken together, these data provide strong evidence for the occurrence of system(s) enabling the direct incorporation of ADPG into the *E*. *coli* cell.

### ADP released by glycogen synthase from ADPG entering the cell can feed the adenine nucleotide pool

**Figure 2:**
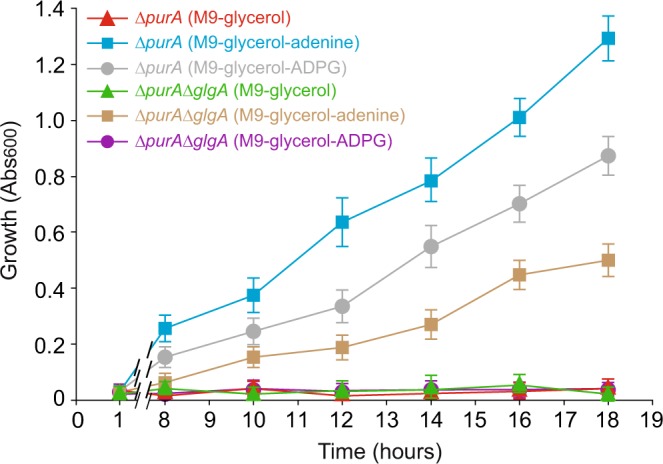
ADP released by GlgA from ADPG entering the cell can feed the adenine nucleotide pool. Growth of Δ*purA* and Δ*purA*Δ*glgA* cells cultured in liquid M9 medium supplemented with 2% (v/v) glycerol (M9-glycerol) with or without 100 μM adenine or ADPG supplementation. Values represent means ± SE obtained from four independent experiments with 3 replicates for each experiment.

Unlike exogenously added adenosine, AMP and ADP (all compounds that can potentially be generated from enzymatic breakdown of ADPG in the *E*. *coli* periplasm), external ADPG could not sustain BW25113 growth when provided on minimal culture medium as the only carbon source (Supplementary Fig. S2). Although these findings added further evidence that *E*. *coli* BW25113 is capable of directly incorporating ADPG, they also argued against a role of the observed ADPG uptake for catabolic purposes only, whereby the sugar moieties and amino group of this compound could be made available for bacterial growth^17^. We thus tested the possibility that the incorporation of ADPG and its subsequent use by GlgA for glycogen synthesis could form part of an adenine salvage pathway, provided that this step also releases ADP that can be funneled into the metabolism of adenine nucleotides^17^. Towards this end, we compared the growth profiles of *∆purA* mutant cells impaired in the IMP-to-AMP conversion biosynthetic pathway^17^ and *∆purA∆glgA* double mutant (additionally lacking GlgA and thus unable to produce ADP from ADPG) on liquid minimal M9 medium supplemented with 2% (v/v) glycerol (M9-glycerol) as the carbon and energy source. As expected, *∆purA* cells showed impaired growth in M9-glycerol, but could resume grow after supplementation with adenine (Fig. 2). Notably, *∆purA* cells, but not *∆purA∆glgA* cells, could grow in M9-glycerol-ADPG medium (Fig. 2), which strongly supports the above hypothesis that ADPG imported from the extracellular medium, followed by the release of ADP by GlgA, is part of an adenine scavenging/salvage pathway capable of feeding the purine nucleotides metabolism in *E*. *coli*.

### Genome-wide screening of genes affecting glycogen synthesis from imported ADPG in *E*. *coli* cells

To identify structural and regulatory components of the machinery involved in the direct incorporation of extracellular ADPG into *E*. *coli* cells, we screened the Keio collection of single-gene deletion mutants for altered glycogen content using the iodine staining technique. An inspection of the 3,985 mutants in the collection revealed 13 mutants (0.3% of the library) with a yellowish, “glycogen-deficient” iodine staining phenotype and 72 mutants (1.8% of the library) with a brown, “glycogen-excess” iodine staining phenotype when cultured in solid KM-ADPG. The 85 genes whose deletions affected glycogen accumulation were classified into clusters of orthologous groups (COGs). Tables 1 and 2 show the genes whose deletion(s) lead to either glycogen-deficient/less or glycogen-excess phenotypes, respectively, and Supplementary Tables 1 and 2 describe the function of each gene product. The clustering indicates that, in *E*. *coli* BW25113, the synthesis of glycogen from ADPG imported from the extracellular medium is affected by functions that fit into three major groups: (i) carbon sensing and metabolism; (ii) nucleoside transport; and (iii) envelope composition and integrity. In the following sections we discuss the roles of some of the identified functions.

**Table 1:** Genes that, when deleted, cause a “glycogen-deficient” or “glycogen-less” phenotype in *E*. *coli* cells cultured in solid KM-ADPG.

COG category	Genes
**Metabolism**	
C. Energy production and conversion	*lpd*
F. Nucleotide transport and metabolism	*cya*, *nupC*, *nupG*
G. Carbohydrate transport and metabolism	*glgA*, *glgC*
**Cellular processes**	
T. Signal transduction mechanisms	*crp*
**Poorly characterized**	
R. General function prediction only	*yedF*, *yegV*
S. Function unknown	*rssA*, *ybjL*, *ycgB*, *yoaE*

Genes are classified into cluster of orthologous groups (COG) categories.

**Table 2:** Genes that, when deleted, cause a “glycogen-excess” phenotype in *E*. *coli* cells cultured in solid KM-ADPG.

COG category	Genes
**Metabolism**	
C. Energy production and conversion	gpmM*
E. Amino acid transport and metabolism	*carA*, *carB*, *proA*, *ydgI*
G. Carbohydrate transport and metabolism	pgm**, galU**
H. Coenzyme transport and metabolism	*ygfA*
I. Lipid transport and metabolism	fabH**, *prpE*, ybgC*
P. Inorganic ion transport and metabolism	*narU*, phoP**, phoU*, pstA*, pstC*
**Cellular processes**	
D. Cell cycle control, cell division, chromosome partitioning	dedD**, envC**
M. Cell wall/membrane/envelope biogenesis	amiD*, galU**, envZ*, gmhB**, ldcA**, lpcA**, lpxL**, mltE*, mrcB**, nlpI**, ompA**, ompC**, pal**, *proX*, rfaC**, rfaE**, rfaF**, rfaP**, tolB**, tolQ**, tolR**, ycfM**, yciM**, yciS**
O. Post-translational modification, protein turnover, chaperones	clpA*, degP**, dsbD*, *prc*, *proQ*, surA**
T. Signal transduction mechanisms	*cheB*, *hflD*, pphA*
U. Intracellular trafficking, secretion and vesicular transport	tatA**, tatB**, tatC**
V. Defense mechanisms	*emrE*
**Information**, **storage and processing**	
J. Translation, ribosomal structure and biogenesis	*prfB*, *rpsT*
K. Transcription	rfaH**, rseA**, *ydaS*
L. DNA replication, recombination and repair	dam**, *dnaT*, *recB*, ruvC*, xerC*, xerD*
**Poorly characterized**	
R. General function prediction only	*ygeG*, *yhcB*, *yhdP*
S. Function unknown	slyB*, *yfgJ*, *yigZ*, *yiiS*

Genes are classified into COG categories. Asterisks indicate genes that have been shown to (**) or are suspected to (*) participate in the biogenesis/integrity of envelope components.

#### Carbon sensing and metabolism

**Figure 3:**
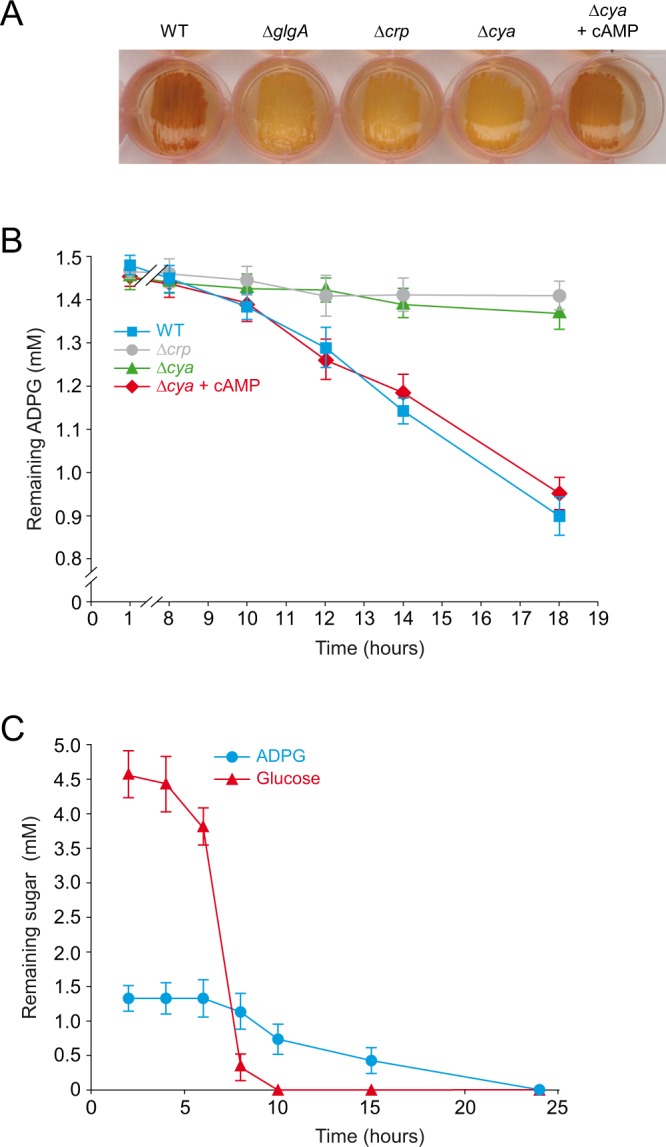
ADPG incorporation into *E*. *coli* cells is regulated by the cAMP/CRP system. (**A**) Glycogen iodine staining of BW25113 WT, *∆glgA*, *∆crp* and *∆cya* cells cultured in solid KM supplemented with 1.5 mM ADPG. (**B**) Time-course analysis of ADPG consumption by BW25113 (WT), *∆crp* and *∆cya* cells cultured in liquid M9 medium supplemented with 2% (v/v) glycerol and 1.5 mM ADPG (M9-glycerol-ADPG). Growth curves are shown in Supplementary Fig. S3B. (**C**) ADPG and glucose consumption by BW25113 WT cells cultured in liquid M9 medium supplemented with 5 mM glucose and 1.5 mM ADPG. Growth curves are shown in Supplementary Fig. S3C. In (**A**,**B**), *∆cya* cells were cultured with or without 1 mM cAMP supplementation. In (**B**,**C**) values represent means ± SE obtained from four independent experiments with 3 replicates for each experiment.

As expected, *∆glgA* displayed a yellow iodine staining phenotype when cultured in KM-ADPG **(**Table 1). ∆*glgC* cells also accumulated low glycogen, which can be ascribed to the polar effect that *glgC* deletion has on the expression of the downstream *glgA* gene^9^. Notably, both *∆crp* and *∆cya* cells displayed a yellow iodine staining phenotype (Table 1, Fig. 3A), indicating that the extracellular ADPG-to-glycogen conversion process is under positive cAMP/CRP control. This inference was corroborated by the analysis of the glycogen content in *∆cya* cells cultured in KM-ADPG supplemented with cAMP. Under these conditions, *∆cya* cells displayed a brown (WT) iodine staining phenotype (Fig. 3A). Since *glgBXCAP* operon expression is not subject to cAMP/CRP control^6,9^, these results strongly suggested that the low glycogen content witnessed in *∆crp* and *∆cya* cells could be a consequence of reduced ADPG incorporation rather than decreased expression of glycogen genes. To test this hypothesis we carried out time-course analyses of ADPG consumption by measuring the ADPG remaining in the culture medium of BW25113 WT, *∆crp* and *∆cya* cells grown in liquid minimal M9-ADPG medium supplemented with either glycerol (a carbon source unable to induce CCR) or glucose (a CCR-inducing carbon source). We found that ADPG levels gradually declined in the WT cell cultures grown in M9-glycerol-ADPG medium (Fig. 3B). In contrast, *∆crp* and *∆cya* cells did not consume ADPG even after prolonged incubation in the same medium (Fig. 3B). Notably, ADPG consumption measured in *∆cya* cultures reverted to that of WT cultures when cAMP was included in the culture medium (Fig. 3B). Furthermore, the consumption of ADPG by WT cells grown in M9-glucose-ADPG medium was observed only when the glucose had almost disappeared from the culture medium (Fig. 3C). The overall data strongly indicated that *E*. *coli* possesses a cAMP/CRP-regulated machinery that enables the cell to take up extracellular ADPG.

#### Nucleoside transport

**Figure 4:**
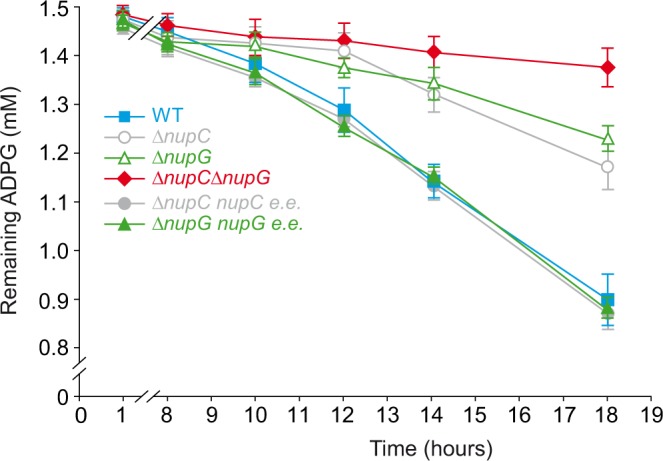
The inner membrane NupC and NupG transporters are the two major transport systems involved in the incorporation of ADPG in *E*. *coli*. Time-course analysis of ADPG consumption by WT, *∆nupC*, *∆nupC* ectopically expressing *nupC* (*∆nupC nupC e*.*e*.*)*, *∆nupG*, *∆nupG* ectopically expressing *nupG* (*∆nupG nupG e*.*e*.), and *∆nupC∆nupG* cells cultured in liquid M9 medium supplemented with 2% (v/v) glycerol and 1.5 mM ADPG. Values represent means ± SE obtained from four independent experiments with 3 replicates for each experiment. Growth curves are shown in Supplementary Fig. S3D.

We noticed that *∆nupC* and *∆nupG* cells accumulated low glycogen (Table 1). The *E*. *coli* NupG and NupC inner membrane proteins are proton motive force-driven transporters that participate in nucleoside scavenging for the synthesis of nucleotides and deoxynucleotides via salvage pathways^17–19^. To investigate the possible involvement of these transporters in the incorporation of extracellular ADPG in *E*. *coli*, we conducted time-course ADPG consumption analyses in WT, *∆nupG*, *∆nupC* and *∆nupG∆nupC* cells grown in liquid M9-glycerol-ADPG medium. As shown in Fig. 4, the ADPG remaining in the culture medium decreased more slowly in both *∆nupG* and *∆nupC* cell cultures than in WT cultures. The observed reduction in ADPG consumption was even more drastic in *∆nupG∆nupC* double-mutant cultures (Fig. 4). The overall data strongly indicated that NupG and NupC are the two major transport systems involved in linking the uptake of extracellular ADPG with glycogen biosynthesis in *E*. *coli* cells.

We also observed that Δ*lpd* mutant cells accumulated low levels of glycogen (Table 1). This mutant lacks lipoamide dehydrogenase, a component of the pyruvate dehydrogenase and 2-oxoglutarate dehydrogenase complexes catalyzing the oxidative decarboxylation of pyruvate and 2-oxoglutarate, respectively, using NAD^+^ as co-substrate. Since the NADH produced by these enzymes feeds the aerobic electron transport chain that generates the H^+^ gradient and membrane potential required for substrate import, it is conceivable that the activities of H^+^/substrate symporters such as NupC and NupG would be affected in the Δ*lpd* mutant thus reducing ADPG import and explaining the lower accumulation of glycogen observed (Table 1).

#### Envelope composition and integrity

**Figure 5:**
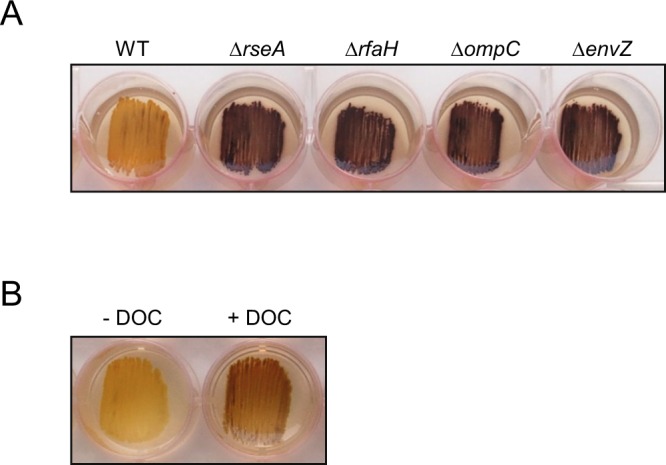
Extracytoplasmic stress exerts a positive effect on the synthesis of glycogen dependent on extracellular ADPG in *E*. *coli*. Glycogen iodine staining of (**A**) BW25113 WT cells and *∆rseA*, *∆rfaH*, *∆ompC* y *∆envZ* mutants cultured in solid KM-ADPG, and (**B**) BW25113 WT cells cultured in solid KM-ADPG without (−DOC) or with (+DOC) 0.1% (w/v) DOC supplementation.

Remarkably, more than two-thirds of the *E*. *coli* BW25113 genes that, when deleted, resulted in a glycogen-excess phenotype, are directly or indirectly related to the biogenesis and maintenance of cell envelope components, including OM proteins (*e*.*g*. *ompC* and *envZ*) and LPS (*e*.*g*. *rfaH*) (Table 2, Supplementary Table 2, Fig. 5A). The loss of any of these genes, although not sufficient to cause cell death, alters the integrity of the cell envelope, which in *E*. *coli* is monitored by RpoE, an essential sigma factor that responds to the presence of extracytoplasmic unfolded proteins^20–22^. RpoE governs the expression of as many as 200 genes involved in the synthesis, assembly, and homeostasis of the OM proteins and LPSs that are needed to counteract envelope damage^20–22^. Notably, *∆rseA* mutant cells lacking the main RpoE specific antisigma factor RseA cultured in KM-ADPG displayed a “dark brown”, glycogen-excess iodine staining phenotype (Table 2, Fig. 5A).

### Extracytoplasmic stress facilitates the incorporation of extracellular ADPG into *E*. *coli* cells

The results presented above indicated that extracytoplasmic stress exerts a positive effect on the synthesis of glycogen with extracellular ADPG as a precursor. To test this hypothesis, we supplemented KM-ADPG with sodium deoxycholate (DOC), a bile acid that causes envelope injury^23,24^. As shown in Fig. 5B, we found that this agent increases glycogen accumulation in BW25113 cells.

**Figure 6:**
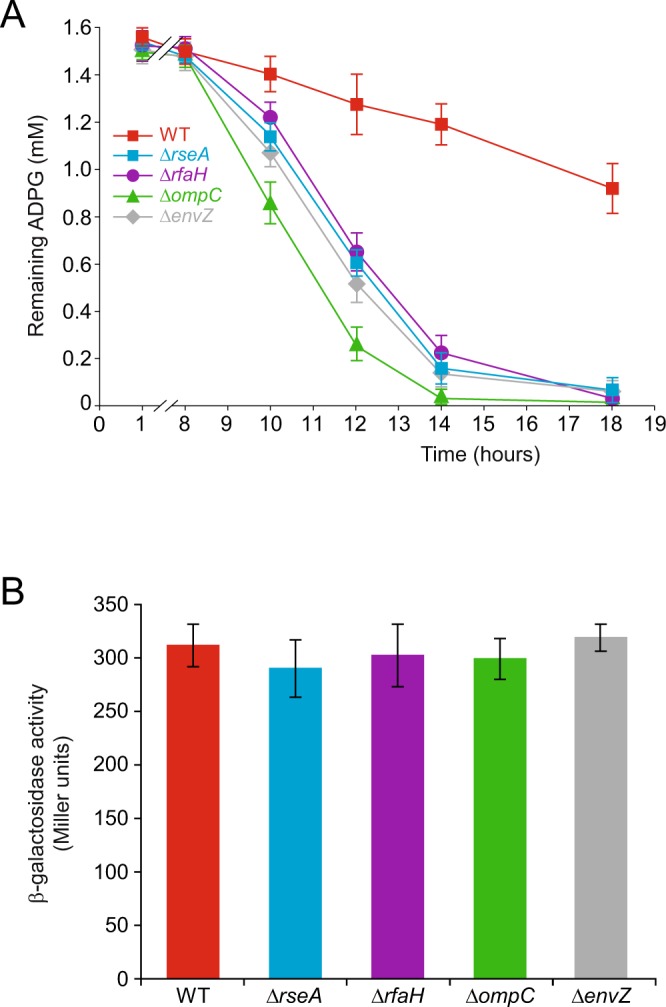
Extracytoplasmic stress exerts a positive effect on the incorporation of ADPG but not on the expression of glycogen genes in *E*. *coli* cells. (**A**) ADPG consumption by BW25113 WT, *∆rseA*, *∆rfaH*, *∆ompC* and *∆envZ* cells cultured in M9 medium supplemented with 2% (v/v) glycerol and 1.5 mM ADPG (M9-glycerol-ADPG). Values represent means ± SE obtained from four independent experiments with 3 replicates for each experiment. Growth curves are shown in Supplementary Fig. S3E. (**B**) Expression of chromosomal *glgB::lacZY* fusion at the onset of the exponential growth phase in BW25113 WT, *∆rseA*, *∆rfaH*, *∆ompC* y *∆envZ* cells cultured in M9-glycerol-ADPG.

The high glycogen content observed in envelope-stressed *E*. *coli* cells cultured in ADPG-containing medium could be ascribed to either enhanced incorporation of ADPG, an augmented expression of glycogen (*glgBXCAP*) genes, or a combination of both. To differentiate between these possibilities, we compared the ADPG consumption and glycogen gene expression in WT and “high-glycogen”, envelope-stressed mutants including *∆rseA*, *∆rfaH*, *∆envZ* and *∆ompC* mutants (cf. Table 2) grown in liquid M9-glycerol-ADPG medium. As shown in Fig. 6A, these analyses revealed that envelope-stressed mutants exhibited higher ADPG consumption than WT cells. Furthermore, glycogen gene expression in these mutants, as judged by the β-galactosidase activity of *glgB::lacZY* fusions measured at the onset of the stationary phase, were equivalent to that of WT cells (Fig. 6B). The overall data strongly support that the high glycogen content observed in envelope-stressed *E*. *coli* cells cultured in ADPG-containing medium mainly stems from enhanced incorporation of extracellular ADPG.

### The cAMP/CRP regulatory system controls the incorporation of extracellular ADPG into envelope stressed cells

**Figure 7:**
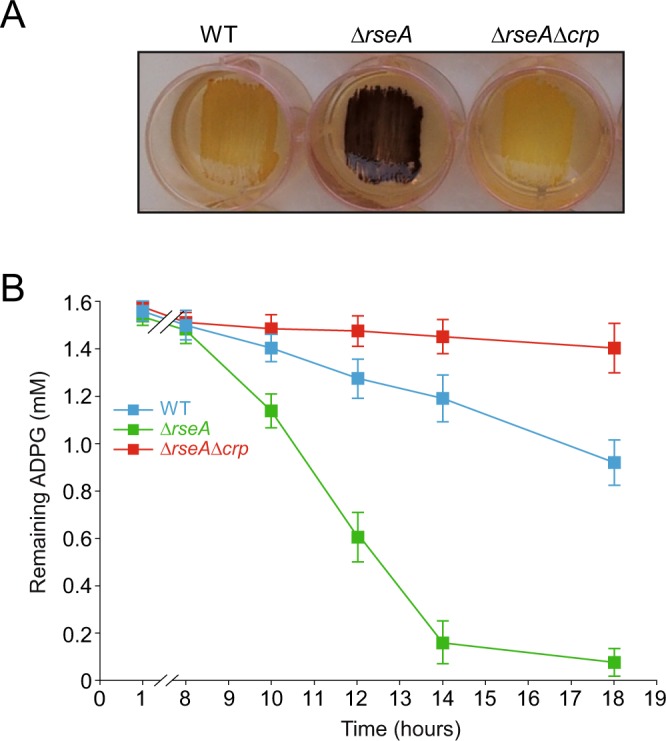
The cAMP/CRP system controls the incorporation of extracellular ADPG into envelope-stressed cells. (**A**) Glycogen iodine staining of BW25113 WT, *∆rseA* and *∆rseA∆crp* cells cultured in solid KM-ADPG. (**B**) ADPG consumption by BW25113 WT, *∆rseA* and *∆rseA∆crp* cells cultured in liquid M9 medium supplemented with 2% (v/v) glycerol and 1.5 mM ADPG. Values represent means ± SE obtained from four independent experiments with 3 replicates for each experiment. Growth curves are shown in Supplementary Fig. S3F.

As shown above, cAMP/CRP is an important determinant of ADPG transport into non-stressed *E*. *coli* cells (cf. Fig. 3). To explore whether this regulatory system also controls the incorporation of ADPG into envelope-stressed cells, we compared the glycogen contents in WT, *∆rseA* and *∆rseA∆crp* cells cultured in solid KM-ADPG. We also conducted time-course ADPG consumption analyses when these cells were cultured in liquid M9-glycerol-ADPG. Notably, transduction of the *∆crp* allele into a *∆rseA* mutant background not only reverted the “glycogen-excess” phenotype of KM-ADPG grown *∆rseA* cells (Fig. 7A), but also strongly decreased ADPG consumption (Fig. 7B). These data thus strongly indicated that ADPG incorporation and use in glycogen synthesis in envelope-stressed cells involves cAMP/CRP regulated transport system(s).

### NupC and NupG are the main transport systems involved in the incorporation of extracellular ADPG into envelope-stressed *E*. *coli* cells

**Figure 8:**
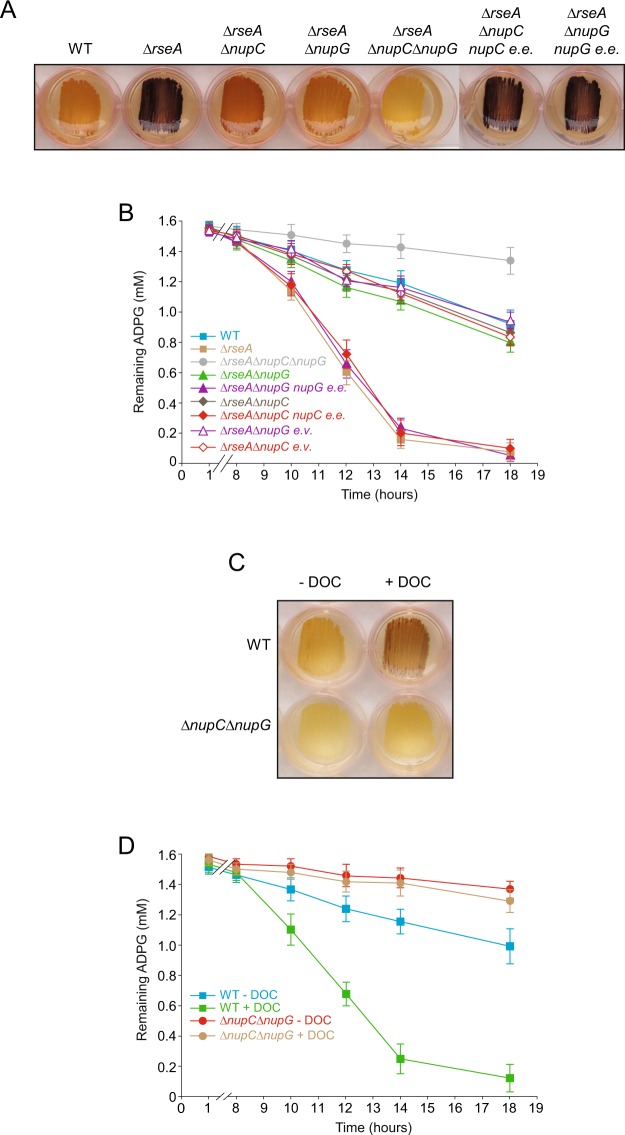
NupC and NupG are the main transport systems involved in the incorporation of extracellular ADPG into envelope stressed *E*. *coli* cells. (**A**) Glycogen iodine staining of *∆rseA*, *∆rseA∆nupC*, *∆rseA∆nupG* and *∆rseA∆nupC∆nupG* cells, along with *∆rseA∆nupC* cells ectopically expressing *nupC* and *∆rseA∆nupG* cells ectopically expressing *nupG* (*∆rseA∆nupC nupC e*.*e*. and *∆rseA∆nupG nupG e*.*e*., respectively) cultured in solid KM-ADPG. (**B**) ADPG consumption by BW25113 WT, *∆rseA*, *∆rseA∆nupC*, *∆rseA∆nupC* bearing the empty pSU18 vector, *∆rseA∆nupG*, *∆rseA∆nupG* bearing the empty pSU18 vector, *∆rseA∆nupC∆nupG*, *∆rseA∆nupC nupC e*.*e*., *∆rseA∆nupG nupG e*.*e*., cells cultured in liquid M9-glycerol-ADPG medium. Growth curves are shown in Supplementary Fig. S3G. (**C**) Glycogen iodine staining of WT and *∆nupC∆nupG* cells cultured in solid KM-ADPG with or without 0.1% (w/v) DOC supplementation. (**D**) ADPG consumption by BW25113 WT and *∆nupC∆nupG* cells cultured in liquid M9-glycerol-ADPG medium with or without 0.1% (w/v) DOC supplementation. Growth curves are shown in Supplementary Fig. S3H. In (**B**,**D**) values represent means ± SE obtained from four independent experiments with 3 replicates for each experiment.

NupG and NupC are major determinants of extracellular ADPG incorporation in non-stressed *E*. *coli* cells (cf. Fig. 4). Both *nupC* and *nupG* are positively regulated by cAMP/CRP^6,17^ which, in principle, would suggest that the cAMP/CRP-regulated conversion of extracellular ADPG into glycogen in envelope-stressed cells involves NupC and NupG. To test this possibility, we compared the glycogen contents in WT, *∆rseA*, *∆rseA∆nupG*, *∆rseA∆nupC* and *∆rseA∆nupC∆nupG* cells cultured in solid KM-ADPG. We also conducted time-course ADPG consumption analyses when these cells were grown in liquid M9-glycerol-ADPG medium. Notably, the transduction of *∆nupG* or *∆nupC* null alleles into a *∆rseA* mutant background reversed, to a significant extent, the “glycogen-excess” (Fig. 8A) and “rapid ADPG consumption” (Fig. 8B) phenotypes of *∆rseA* cells. This effect was even more drastic when the *∆nupG* and *∆nupC* null alleles were simultaneously incorporated into *∆rseA* cells (Fig. 8A,B). In addition, the expression of *nupC* and *nupG* from plasmids in *∆rseA∆nupC* and *∆rseA∆nupG* double mutants, respectively, considerably increased the glycogen contents and ADPG consumption (Fig. 8A,B).

We also compared the glycogen contents in WT and *∆nupC∆nupG* cells cultured in KM-ADPG medium with or without DOC supplementation, and conducted time-course ADPG consumption analyses when these cells were grown in liquid M9-glycerol-ADPG medium. The results showed that, unlike in WT cells, DOC treatment in *∆nupC∆nupG* cells did not increase glycogen accumulation (Fig. 8C) or ADPG consumption (Fig. 8D).

## Discussion

In this work we have demonstrated that *E*. *coli* is capable of directly incorporating ADPG from the external medium through the inner-membrane NupG and NupC transporters (Figs 1, 4 and 8). ADPG uptake by *E*. *coli* cells was triggered by the deficiency of a CCR-inducing, rapidly metabolizable primary carbon source (Fig. 3C), and facilitated under conditions of envelope stress (Figs 5 and 6). In *E*. *coli*, different broad regulatory systems are responsible for resolving situations of nutrient deficiency and membrane stress, including the cAMP/CRP system, the RpoS-dependent general stress response, the ppGpp-mediated stringent response and the RpoE-dependent envelope stress response^5,7,20–22,25–28^. The results presented in Figs 3 and 7 showing that *∆crp* or *∆cya* mutants are not capable of incorporating extracellular ADPG both in non-stressed and in envelope-stressed cells indicated that the cAMP/CRP system is a major regulatory player in either condition. To our knowledge this is the first report showing the capacity of a bacterial species of directly taking up ADPG from the external medium, also identifying the responsible mechanisms and regulatory components of this process.

*E*. *coli* is the predominant facultative anaerobe of the commensal microbiota of the mammalian intestine, a complex ecosystem largely dominated by obligate anaerobes of the *Firmicutes* and *Bacteroidetes* phyla^1,2,29,30^. The *E*. *coli* population of the large intestine resides within the mucus layer lining the epithelium as part of a complex and highly competing microbial community^1,2,29,30^. The relative low representation of *E*. *coli* in the microbiota provides a measure of the undergoing intense competition for resources, and supports the notion that the population of this organism transits the intestine mostly on a hunger/scavenging lifestyle^2,29–31^. It follows that any specific capability of this species to obtain advantages of the prevailing conditions, such as the use of compounds in low demand due to the requirement of specific transporters and/or metabolic pathways not widely available among its competitors, may provide a significant fitness advantage and even define a new ecological niche for particular subspecies^2^.

*E*. *coli* survival strategies include an increased metabolic versatility allowing the uptake and utilization of different carbon sources, proper tuning of nutrient and other stress responses, tolerance mechanisms for environmental aggressors, adaptive anticipation of environmental changes, rapid selection of compensating mutations upon loss of an important function, etc.^2–7,24–38^. In the specific case of glycogen metabolism, it has been shown for both commensal (K-12) and pathogenic (O157:H7) *E*. *coli* strains that deletion of the *glgA* or *glgP* genes result in significant colonization defects of the mouse intestine^29^. These observations led to proposals that glycogen accumulation during occasional excess of nutrients may provide a fitness advantage for growth during more common phases of hunger^2,29^. The results of this work complement this view.

The diets of many mammals include starchy plant organs possessing relatively elevated levels of ADPG^13,14^. The ability of *E*. *coli* to scavenge and use this compound for glycogen synthesis (this work) may certainly provide several advantages in the highly competitive environment of the large intestine. First, glycogen biosynthesis employing ADPG produced internally from the GlgC-mediated reaction with G1P and ATP as substrates imposes a relatively high energy burden on the cell^8^, and hence it is conceivable that the uptake of extracellular ADPG for this purpose will be advantageous under the carbon and energy limiting conditions of this environment. Second, the observation that the ADP generated from the imported ADPG by GlgA action may also feed the adenine nucleotides pool (Fig. 2) would certainly represent another substantial benefit considering that it bypasses most of the ATP-demanding reactions of the *de novo* adenine biosynthetic pathway^17^.

The *E*. *coli* OM may pose restrictions to the access of ADPG to the inner membrane-located NupC and NupG transporters, providing that facilitated diffusion of this compound to the periplasm most likely occurs through the general porins or the Tsx nucleoside channel^17,20,39^. The *E*. *coli* population residing in the intestine is exposed to several membrane-damaging substances occurring in the bile that affect OM integrity and compromise its permeability barrier functions^24^. Remarkably, results presented in this work have shown that alterations of the *E*. *coli* envelope integrity either by mutation or by the presence of the bile acid DOC in the growth medium resulted in an increased ADPG uptake with concomitant increments in glycogen accumulation by the cells (Figs 5 and 6). It is thus conceivable that a limited envelope stress response during transit of *E*. *coli* through the intestine facilitates the uptake of ADPG, which could be employed intracellularly by GlgA for glycogen synthesis and adenine nucleotides provision. This may represent another example of bacterial adaptation to a natural habitat of slow growth exerted by a limited availability of nutrients and/or aggressive environmental conditions, in which the exposure of cells to envelope-damaging substances and the concomitant stress response paves the way for an increased uptake of compounds such as ADPG serving purposes in survival and growth.

**Figure 9:**
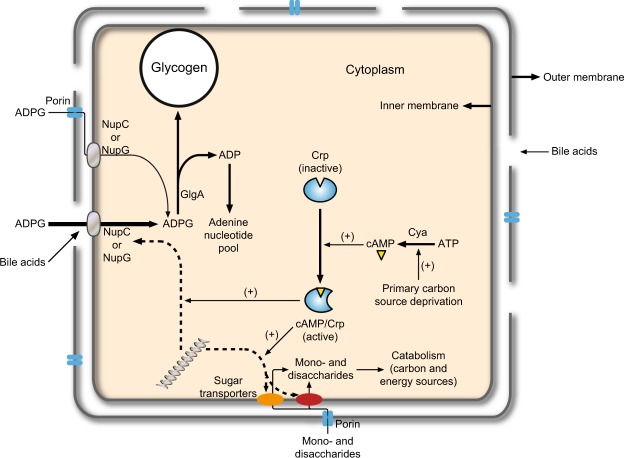
Suggested model for the regulation of extracellular ADPG uptake and utilization in *E*. *coli* in the mammalian intestine. According to this model, NupC and NupG, as well as the integrity of the outer membrane (OM), act as major determinants of the process. Under conditions of glucose (primary carbon source) limitation, augmented cAMP levels and concomitant activation of CRP will enhance the expression of *nupC* and *nupG*, thus favoring the scavenging of extracellular ADPG. These conditions will also enhance the expression of pathways involved in the catabolism of mono- and disaccharides derived from the degradation of complex polysaccharides by the intestine microbiota. It is conceivable that the OM of *E*. *coli* will be periodically damaged by bile acids and other membrane-damaging substances and conditions occurring in the intestine, allowing passive diffusion of ADPG molecules into the periplasm and access to the inner membrane NupC and NupG transporters. In the cytoplasm, the scavenged ADPG is employed to produce glycogen and ADP by GlgA, the former used as a carbon and energy storage compound for survival and colonization^2,29^ and the latter to fuel purine nucleotides metabolism (this work).

Figure 9 illustrates a suggested model for the regulation of the uptake and utilization of extracellular ADPG by *E*. *coli* in the large intestine, in which cAMP/CRP-regulated NupC and NupG, as well as the integrity of the OM, act as major determinants of the process. When *E*. *coli* cells face limitation of the rapidly metabolizable primary carbon source glucose (as is likely the case during intestine transit), the consequent augmentation of cAMP levels and concomitant activation of CRP will enhance the expression of *nupC* and *nupG* and genes that code for transporters and pathways involved in the catabolism of “secondary” carbon sources available in this environment^2,5–7,17^. Under the harsh conditions of the intestine, it is conceivable that the OM of *E*. *coli* will be periodically injured, thus compromising its permeability barrier functions and allowing the passive diffusion of ADPG into the periplasm and access to the NupC and NupG transporters. Once in the cytoplasm the scavenged ADPG molecule can be used by GlgA to synthesize glycogen with the release of ADP^8^, with glycogen serving as a carbon and energy storage compound^2,29^, and ADP feeding the adenine nucleotides pool as we show in this work.

Adaptation and speciation of prokaryotes is fostered by ecological divergence combined with their rapid propensity both to amass novel gene loci by horizontal gene transfer and/or evolve novel functions by point mutations^33^. It is thus tempting to speculate that a new function derived from mutations at *nupC* and *nupG* facilitating the incorporation of ADPG was selected in an ancestor of the *Enterobacteriales*^10^ adapting to a host feeding on starchy plant tissues^10^. Similar type of mutations enabling the incorporation of ADPG may have been selected in other bacteria residing in close contact with starchy organs of plants accumulating ADPG.

## Methods

### Bacterial strains and plasmids

The genotypes of the *E*. *coli* strains, mutants and plasmids used in this work are shown in Table 3. *E*. *coli* K-12 derivative BW25113 single-gene knockout mutants were obtained from the Keio collection^15^. Double and triple knockout mutants were constructed using single knockout mutants from the Keio collection. The kanamycin resistance cassette was removed from the recipient strain using the temperature-sensitive plasmid pCP20 carrying the FLP recombinase^40^. The deletion from the donor strain was then P1-transduced into the recipient strain. Kanamycin-containing LB plates were used to select the double and triple mutants, after which deletions were verified by PCR. To produce cells expressing *nupC* and *nupG* in trans, *nupC* and *nupG* and their promoter sequences were amplified by PCR using the chromosomal DNA of *E*. *coli* BW25113 as a template and the following primers: *nupC* forward: 5′-CGCGGATCCTGTATGACAGAT-3′; *nupC* reverse: 5-AACTGCAGTTACAGCACCAGT-3; *nupG* forward: 5′-CGCGGATCCCTCAGGGGCAAA-3′; *nupG* reverse: 5′ AACTGCAGTTAGTGGCTAACC-3′.

**Table 3:** *E*. *coli* strains and plasmids used in this work.

Designation	Description	Source
**Bacteria**		
TG1	K-12 strain; *supE thi-1* Δ*(lac-proAB)* Δ*(mcrB-hsdSM)5*, *(rK*^−^*mK*^−^) F′ [*traD36 proAB* + *lacIq lacZΔM15*]	^45^
BL21	B-strain; F− ompT gal dcm lon hsdSB(rB−mB−) [malB+]K-12(λS)	
BW25113	*lacI*^q^ *rrnB*_T14_ Δ*lacZ*_WJ16_ *hsdR514* Δ*araBAD*_AH33_ Δ*rhaBAD*_LD78_	Keio collection^15^
AC70R1-504	B-strain, F^−^ *glgQ*^−^ *glgC*^−^ *glpD*^−^	^16^
Δ*glgA*	BW25113 Δ*glgA*::Km^R^	Keio collection^15^
Δ*nupC*	BW25113 Δ*nupC*::Km^R^	Keio collection^15^
ΔnupC*	BW25113 Δ*nupC*::Km^R^ where Km^R^ was removed using FRT sites	This work
Δ*nupG*	BW25113 Δ*nupG*::Km^R^	Keio collection^15^
ΔnupG*	BW25113 Δ*nupG*::Km^R^ where Km^R^ was removed using FRT sites	This work
Δ*crp*	BW25113 Δ*crp*::Km^R^	Keio collection^15^
Δcrp*	BW25113 Δ*crp*::Km^R^ where Km^R^ was removed using FRT sites	This work
Δ*cya*	BW25113 Δ*cya*::Km^R^	Keio collection^15^
Δ*rfaH*	BW25113 Δ*rfaH*::Km^R^	Keio collection^15^
ΔrfaH*	BW25113 Δ*rfaH*::Km^R^ where Km^R^ was removed using FRT sites	This work
Δ*ompC*	BW25113 Δ*ompC*::Km^R^	Keio collection^15^
ΔompC*	BW25113 Δ*ompC*::Km^R^ where Km^R^ was removed using FRT sites	This work
Δ*envZ*	BW25113 Δ*envZ*:Km^R^	Keio collection^15^
ΔenvZ*	BW25113 Δ*envZ*::Km^R^ where Km^R^ was removed using FRT sites	This work
Δ*rseA*	BW25113 Δ*rseA*::Km^R^	Keio collection^15^
ΔrseA*	BW25113 Δ*rseA*::Km^R^ where Km^R^ was removed using FRT sites	This work
Δ*rseA*Δ*crp*	BW25113 Δ*rseA* P1 phage transduced in Δcrp*	This work
Δ*rseA*Δ*nupC*	BW25113 Δ*rseA* P1 phage transduced in ΔnupC*	This work
Δ*rseA*Δ*nupG*	BW25113 Δ*rseA* P1 phage transduced in ΔnupG*	This work
Δ*nupC*Δ*nupG*	BW25113 Δ*nupC* P1 phage transduced in ΔnupG*	This work
Δ*rseA*Δ*nupC*Δ*nupG*	BW25113 Δ*nupC*Δ*nupG* where complete *rseA* was replaced by Sp^R^ cassette	This work
*glgB::lacZY*	BW25113 *glgB*::*lacZY* transcriptional fusion	^12^
Δ*ompC glgB*::*lacZY*	BW25113 *glgB*::*lacZY* transcriptional fusion in ΔompC*	This work
Δ*envZ glgB*::*lacZY*	BW25113 *glgB*::*lacZY* transcriptional fusion in ΔenvZ*	This work
Δ*rfaH glgB*::*lacZY*	BW25113 *glgB*::*lacZY* transcriptional fusion in ΔrfaH*	This work
Δ*rseA glgB*::*lacZY*	BW25113 *glgB*::*lacZY* transcriptional fusion in ΔrseA*	This work
**Plasmids**		
pCP20	Plasmid expressing FLP recombinase, Amp^R^, used for removal of Km^R^ cassettes	^46^
pSU18	Expression plasmid, Cm^R^	^41^
pSU18-*nupC*	pSU18 directing *nupC* expression	This work
pSU18-*nupG*	pSU18 directing *nupG* expression	This work

Amp^R^, ampicillin resistance; Km^R^, kanamycin resistance; Cm^R^, chloramphenicol resistance.

The amplified products were digested with BamHI and PstI and the resulting fragments were ligated to the corresponding restriction sites in the pSU18 vector^41^ to produce pSU18-*nupC* and pSU18-*nupG*. Bacteria were then transformed with these plasmids using electroporation.

### *lacZY* transcriptional fusions

The kanamycin-resistance cassette of *ΔglgB* cells from the Keio collection^15^ was removed by using a temperature-sensitive plasmid pCP20 carrying the FLP recombinase^40^. The scar sequence left after removal of the resistance cassette included a 34-nucleotide FRT site^15^, which was used to build the *glgB:lacZY* transcriptional fusion as reported by^42^. Briefly, *ΔglgB* cells from the Keio collection carrying a pCP20 plasmid were transformed with pKG137, which has a functional *lacZY* and Km^R^ cassette that was integrated with proper orientation at the FRT site using FLP recombinase. This yielded the *glgB:lacZY* transcriptional fusion where the original resistance cassette of the Keio collection was previously placed. Transcriptional fusions were P1-transduced^43^ into different mutants as necessary. The fusion was verified by PCR using an oligonucleotide (5′-TTCAGGCTGCGCAACTGTTGG-3′) that anneals within *lacZ* (+150 bp reverse orientation) and oligonucleotides that specifically anneal at positions 500 bp upstream of the insertion point. The *glgB:lacZY* fusion yielded a ca. 750 bp PCR amplification product.

### Estimation of cell glycogen content by iodine staining

Cells were grown overnight at 37 °C in plates containing solid KM (1.1% K_2_HPO_4_, 0.85% KH_2_PO_4_, 0.6% Difco yeast extract, 1.8% Difco bacteriological agar) supplemented with the indicated compounds. Glycogen accumulation in the cells was estimated by the iodine staining method^12^ at least in triplicate. The screening for mutants with altered glycogen on the Keio collection^15^ was performed on 96-well plates containing solid KM supplemented with 1.5 mM ADPG. The indicated deletions in mutants with altered glycogen content were further confirmed by PCR using specific primers. To study how extracytoplasmic stress affects glycogen synthesis in the presence of externally added ADPG, cells were grown overnight at 42 °C in plates containing solid KM-ADPG with or without 0.1% (w/v) DOC supplementation.

### Determination of ADPG consumption

ADPG consumption by *E*. *coli* cells was investigated by measuring the ADPG remaining in the culture medium of 2 mL cell cultures growing at 37 °C with rapid gyratory shaking in M9 liquid medium (95 mM Na_2_HPO_4_/44 mM KH_2_PO_4_/17 mM NaCl/37 mM NH_4_Cl/0.1 mM CaCl_2_/2 mM MgSO_4_) supplemented with 2% (v/v) glycerol and 1.5 mM ADPG, unless otherwise indicated. The cultures were started by inoculating overnight culture in LB liquid medium into fresh M9 minimal medium (1:100 v/v) containing the additional compounds indicated in the text. At the indicated culture times, aliquots were withdrawn for growth estimations and the determination of the ADPG remaining in the culture medium. For studies on the effect of extracytoplasmic stress on ADPG uptake, cells were cultured at 42 °C in liquid M9-glycerol-ADPG supplemented with 0.1% (w/v) DOC.

### Analytical procedures

Bacterial growth was followed spectrophotometrically by measuring the absorbance at 600 nm. β-galactosidase activity was measured as described by^44^. To measure the content of nucleotide-sugars remaining in the culture medium, the cultures were centrifuged at the indicated times at 4,400 × g for 15 min, after which the supernatants were collected and heated at 95 °C for two minutes. The content of nucleotide-sugars was measured by HPLC using a system obtained from Waters (Waters Corporation, Milford, MA) fitted with a Partisil-10-SAX column (Sigma Aldrich, St. Louis, MO). Glucose content in the supernatants was measured by HPLC with pulsed amperometric detection using a DX**-** 500 Dionex system (Dionex, Sunnyvale, CA) fitted with a CarboPac PA10 column.

### Statistical analysis

The data presented are the means (±SE) from four independent experiments, with 3–5 replicates for each experiment. The significance of differences between control and mutant lines was statistically evaluated with Student’s t-test using SPSS software. Differences were considered significant if P < 0.05. To evaluate the lack of any meaningful differences in β-galactosidase activity, we calculated 95% confidence intervals for the difference of the mean activity for each of the mutants relative to the wild type, and found that all fell entirely within the region of practical equivalence (75 Miller units).

## Electronic supplementary material

Supplementary Information

## Data Availability

All data generated or analysed during this study are included in this published article and its Supplementary Information files.
