# Supplementary material for: A cAMP/CRP-controlled mechanism for the incorporation of extracellular ADP-glucose in Escherichia coli involving NupC and NupG nucleoside transporters
Source: Sci Rep. 2018 Oct 19;8:15509. doi: 10.1038/s41598-018-33647-w (PMC6195507; doi:10.1038/s41598-018-33647-w)
Supplement: Supplementary file 1 — Supplementary Information [file 41598_2018_33647_MOESM1_ESM.pdf]

SUPPLEMENTARY INFORMATION

Title: A cAMP/CRP-controlled mechanism for the incorporation of extracellular ADP-glucose in *Escherichia coli* involving NupC and NupG nucleoside transporters

Authors: Goizeder Almagro[1], Alejandro M. Viale[2], Manuel Montero[1], Francisco José Muñoz[1], Edurne Baroja-Fernández[1], Hirotada Mori[3] and Javier Pozueta-Romero[1*]

Supplementary Table 1: *E. coli* genes whose deletions caused "glycogen-deficient" or "glycogen-less" phenotypes in *E. coli* cells cultured in solid KM-ADPG medium. Cellular localization: OM (outer membrane), IM (inner membrane), C (cytoplasm), P (periplasm). The function of each gene for which deletion affects glycogen accumulation was identified by referring to the EchoBASE (http://ecoli-york.org/) and EcoCyc (http://www.ecocyc.org/) databases.

Gene	[a]Cellular Localization	Function
crp	C	Cyclic AMP-activated global transcription factor protein, mediator of catabolite repression
cya	C	Adenylate cyclase, cyclic AMP synthesis
glgA	C	Glycogen synthase
glgC	C	ADPG pyrophosphorylase
nupC	IM	High-affinity transporter of (deoxy) nucleosides except (deoxy) guanosine, (deoxy) inosine, and xanthosine; Nucleoside:H^+ symporter of the Concentrative Nucleoside Transporter (CNT) family
nupG	IM	High-affinity transporter of all natural purine and pyrimidine (deoxy) nucleosides except xanthosine; Nucleoside:H^+ Symporter (NHS) family
lpd	C	Lipoamide dehydrogenase, E3 component of pyruvate and 2-oxoglutarate dehydrogenases complexes. Lpd catalyzes the transfer of electrons to the ultimate acceptor, NAD^+
rssA	C	Predicted phospholipase, patatin-like family. Function unknown
ybjL	IM	Putative AAE family transporter. Function unknown.
ycgB	C	RpoS regulon member. Function unknown
yedF	C	predicted sulfurtransferase (TusA family). Function unknown.
yegV	C	Predicted sugar/nucleoside kinase.
yoaE	IM	Predicted inner membrane protein. Function unknown.

Supplementary Table 2: *E. coli* genes whose deletions caused a "glycogen-excess" phenotype in *E. coli* cells cultured in solid KM-ADPG. Cellular localization: OM (outer membrane), IM (inner membrane), C (cytoplasm), P (periplasm). Asterisks indicate genes for which evidence exists (**) or are suspected to (*) participate in the biogenesis/integrity of envelope components. The function of each gene for which deletion affects glycogen accumulation was identified by referring to the EchoBASE (http://ecoli-york.org/) and EcoCyc (http://www.ecocyc.org/) databases.

**Gene deletion (Predicted impairment in the mutant)**	**(Cell location of the gene product)**	**Function of the gene product according to EcoGene (http://www.ecogene.org/old/index.php) and/or the cited references**
amiD (Envelope integrity)*	OM	*amiD* encodes an OM-anchored lipoprotein with 1,6-anhydro-N-acetylmuramyl-L-alanine amidase activity. Probable roles in peptidoglycan (autolysis) [Typas et al. 2012].
carA (purine/pyrimidine metabolism)	C	Carbamoyl phosphate synthase, small subunit. With *carB* below forms the *carAB* operon participating in pyrimidine nucleotide biosynthesis: UMP, UTP and CTP (RNA synthesis); dCTP and dTTP (DNA synthesis) [Jensen et al 2008].
carB (purine/pyrimidine metabolism)	C	Carbamoyl phosphate synthase, large subunit. With *carA* above forms the *carAB* operon participating in pyrimidine nucleotide biosynthesis.
cheB	C	Chemotaxis MCP protein-glutamate methylesterase
clpA (Envelope integrity)*	C	*clpA* encodes an ATP-dependent molecular chaperone that serves as a substrate-specifying adapter for the ClpP serine protease in the ClpAP and ClpAXP protease complexes. Probable roles in cell division by modulating the levels of FtsZ forming the FtsZ ring at midcell and possibly other downstream cell division components [Camberg et al.

		2011, Williams et al. 2014]
dam<br><br>(DNA replication, recombination and/or repair; Envelope integrity)**	C	*dam* encodes a DNA adenine methyltransferase (Dam) responsible for methylation of nearly all GATC sequences in the *E. coli* chromosome. Δ*dam* mutants display a pleiotropic phenotype (increased mutability, hyperrecombination, increased number of single-strand breaks, and random timing of chromosome replication). Δ*dam* mutants of *Salmonella enterica* show severe defects in envelope composition and integrity and increased susceptibility to bile salts such as deoxycholate [Pucciarelli et al. 2002, Begley et al. 2005].
dedD<br><br>(Envelope integrity)**	P, IM	*dedD* encodes a periplasmic protein anchored to the inner membrane which localizes at the septal ring during the late stages of divisome complex assembly required for OM invagination during cell division. *E. coli* Δ*dedD* mutants show mild defects in division under normal conditions and growth impairments under envelope stress induced by detergents or high temperatures [Murata et al. 2011; Typas et al. 2012, Black et al. 2013; Nicolaes et al. 2014].
degP<br><br>(Envelope integrity)**	P	*degP* encodes a periplasmic serine protease required for the degradation of damaged envelope proteins, primarily OM proteins. Δ*degP* mutants accumulate toxic misfolded outer membrane (OM) proteins promoting induction of the RpoE envelope stress response, and show increased susceptibility to high (45-47 °C) temperature. *degP* is also controlled by the CpxR/CpxA two-component system sensing envelope stress [Ruiz and Silhavy 2005, Murata et al. 2011; Rigel and Silhavy 2012, Ge et al. 2014, Klein et al. 2016].
dnaT<br><br>(DNA replication, recombination and/or repair)	C	*dnaT* encodes a protein participating in DNA replication (primosomal protein I) under stress situations such as the SOS response and exposure to base alkylating agents. *E. coli* Δ*dnaT* cells are susceptible to high hydrostatic pressure suggesting envelope alterations [Rooney et al. 2009, Black et al. 2013].
dsbB<br><br>(Envelope integrity)*	IM	The *dsbB* product is a protein disulfide oxidoreductase that re-oxidizes periplasmic DsbA involved in the oxidative folding of many periplasmic and OM proteins. *dsbA* is controlled by the CpxR/CpxA two-component system sensing envelope stress, and Δ*dsbA* mutants have abnormal levels of many periplasmic and OM proteins inducing envelope stress responses

		[Ruiz and Silhavy 2005, Vertommen et al. 2008, Goemans et al. 2014].
emrE (Osmoprotection)	IM	*emrE* encodes a member of the small multidrug resistance (SMR) protein family participating in the efflux of a broad range of toxic quaternary cationic compounds including osmoprotectants, suggesting roles in *E. coli* osmotic tolerance [Bay and Turner 2012]
envC (Envelope integrity)**	P, OM	*envC* encodes a divisome-associated protein bearing a C-terminal LytM domain specifically activating periplasmic AmiA and AmiB N-acetylmuramyl-L-alanine amidases mediating PG hydrolysis at the septal ring. The Δ*envC* mutant shows increased susceptibility to high (45-47 °C) temperature. Other proteins identified in this screening recruited or interacting with the divisome are DedD, MrcB (PBP1B), the PBP1B activator YcfM (LpoB), and TolQ-R-A-B-Pal Cell Envelope Complex (Tol-Pal complex) proteins [Weiss 2004, Murata et al. 2011; Typas et al. 2012].
envZ (Osmoprotection, Envelope integrity)*	IM	*envZ* encodes an osmosensor histidine-protein kinase/phosphatase of the EnvZ/OmpR two-component system. Regulates the production of outer membrane proteins in response to different stress situations including temperature and osmotic stress [Ruiz and Silhavy 2005, Rigel and Silhavy 2012].
fabH (Envelope integrity)**	C	*fabH* encodes β-ketoacyl-ACP synthase participating in the elongation step of fatty acid biosynthesis, thus generating precursors for the synthesis of phospholipids and lipid A of the lipopolysaccharide (LPS). Imbalances in this synthesis induce the RpoE-mediated response. Accordingly, *E. coli* Δ*fabH* mutants show envelope defects and are unable to regulate cell size in response to nutrient excess [Yao et al. 2012, Klein and Raina 2015].
galU (Envelope integrity)**	C	*galU* encodes (UDP-glucose pyrophosphorylase synthesizing UDP-glucose from glucose 1-phosphate and UTP providing precursors for LPS core components. *E. coli* Δ*galU* mutants produce altered LPS structures lacking all sugars beyond the heptose residues, resulting in envelope integrity defects and increased susceptibility to SDS [Jiang et al. 2010, Nicolaes et al. 2014, Klein and Raina 2015]. *galU* participates in bile resistance in *Vibrio cholerae* [Begley et al. 2005].
gmhB (Envelope	C	*gmhB* encodes D,D-heptose 1,7-bisphosphate phosphatase, an enzyme of the ADP-heptose biosynthesis pathway required for LPS core biosynthesis. The absence of L-glycero-D-manno-heptose in the LPS

integrity)**		dramatically increases bacterial susceptibility to hydrophobic antibiotics and detergents indicating defects in envelope integrity. *gmhB* is required for *E. coli* survival at 45-47 °C [Kneidinger et al. 2002, Murata et al. 2011, Nicolaes et al. 2014]. The *gmhA* gene of the ADP-heptose pathway was also identified in this screening (see below).
gpmM (Envelope integrity)*	C	*gpmI* encodes a 2,3-bisphosphoglycerate-independent phosphoglycerate mutase. The *gpmI* gene is the first gene of the *gpmI-envC-yibQ* operon. *envC*, also identified in this screening (see above) encodes the EnvC factor required for the activation of amidases mediating peptidoglycan hydrolysis at the septal ring. *gpmM* deletion may thus have a polar effect on the transcription of the downstream *envC* gene.
hflD (purine biosynthesis)	IM	The *hflD* gene product downregulates bacteriophage lambda lysogenization. *hflD* is the first gene of the *hflD-purB* operon, with *purB* encoding adenylosuccinate lyase involved in purine synthesis [Jensen et al. 2008]. Thus, *hflD* deletion may have a polar effect on the transcription of the downstream *purB* gene
ldcA (Envelope integrity)**	C	*ldcA* encodes a cytoplasmic L,D-carboxypeptidase releasing terminal D-alanine from L-alanyl-D-glutamyl-meso-diaminopimelyl-D-alanine peptides generated during peptidoglycan turnover. *E. coli* Δ*ldcA* mutants contain high levels of the PG precursor uridine 5'-pyrophosphoryl N-acetylmuramyl-tetrapeptide and lyse at the stationary growth phase, thus indicating envelope defects [Templin et al. 1999].
lpcA (Envelope integrity)**	C	*lpcA/gmhA* encodes sedoheptulose 7-phosphate isomerase catalyzing the isomerization of D-sedoheptulose 7-phosphate into D-glycero-D-manno-heptose 7-phosphate, the first committed step of the ADP-heptose synthesis pathway for LPS biosynthesis. The absence of L-glycero-D-manno-heptose in the LPS is associated with dramatically increased bacterial susceptibility to hydrophobic antibiotics, detergents, and high temperature (45-47 °C) indicating envelope integrity defects [Kneidinger et al. 2002, Murata et al. 2011, Nicolaes et al. 2014]. The *gmhB* gene of this pathway was also identified in this screening (see above).
lpxL (Envelope integrity)**	IM	*lpxL/waaM* encodes a KDO2-lipid IVA lauroyl-ACP acyltransferase involved in the synthesis of lipid A during LPS biosynthesis pathway. Δ*lpxL* mutants show altered cell division, growth, and high-temperature (45-47 ºC) susceptibility indicating envelope integrity defects [Murata et al. 2011].

mltE (Envelope integrity)*	OM	*mltE* encodes lytic murein transglycosylase E cleaving β-1,4 glycosidic bonds between N-acetylglucosamine and N-acetylmuramic acid residues in the peptidoglycan. It has roles in PG hydrolysis (autolysis) [Typas et asl. 2012].
mrcB (Envelope integrity)**	IM	*mrcB* encodes the major bifunctional peptidoglycan synthase PBP1B catalyzing the transglycosylation and transpeptidation of murein precursors. PBP1B is mainly involved in cell division interacting with the divisome complex during the late stages of divisome assembly required for OM invagination during septation. Also identified in this screening are the PBP1B activator YcfM (LpoB), EnvC, DedD, and Tol-Pal proteins, which are recruited or interact with the divisome [Typas et al. 2012].
narU (Severe nutrient starvation)	IM	*narU* encodes a member of the Major Facilitator Superfamily (MFS) involved in nitrate/nitrite transport. NarU confers a selective advantage during severe nutrient starvation or slow growth [Clegg et al. 2006].
nlpI (Envelope integrity)**	OM?	*nlpI* encodes a verified lipoprotein. *E. coli* Δ*nlpI* mutants are osmotically sensitive and form filaments at 42 °C indicating envelope defects. *nlpI* overexpression dramatically alteres *E. coli* normal morphology suggesting roles in cell division [Ohara et al. 1999].
ompA (Envelope integrity)**	OM	*ompA* encodes a main OM protein in *E. coli*. Δ*ompA* mutants show alterations in OM architecture and LPS structure triggering the RpoE response [Ruiz and Silhavy 2005, Rigel and Silhavy 2012, Klein and Raina 2015, Klein et al. 2016]
ompC (Osmoprotec tion, Envelope integrity)**	OM	*ompC* encodes a major non-specific porin of the *E. coli* OM. Δ*ompC* mutants show alterations in OM architecture and LPS structure triggering the RpoE response. Transcription of *ompC* is controlled by a complex regulatory network that includes the EnvZ-OmpR two-component system sensing osmotic stress as well as by the CpxR/CpxA two-component system and RpoE-regulated responses sensing envelope stress [Batchelor et al. 2005, Ruiz and Silhavy 2005, Rigel and Silhavy 2012, Klein and Raina 2015; Klein et al. 2016].
pal (Envelope	OM	*pal* encodes a peptidoglycan-associated OM lipoprotein member of the Tol-Pal complex localizing at the divisome during the late stages of divisome assembly and required for OM invagination during septation. *E. coli* Δ*pal* mutant shows increased susceptibility to high temperature (45-

integrity)**		47 °C) [Murata et al. 2011, Typas et al. 2012]. Other proteins also identified in this screening recruited or interacting with the divisome are EnvC, DedD, MrcB (PBP1B), and YcfM (LpoB).
pgm (Envelope integrity)**	C	*pgm* encodes phosphoglucomutase (PGM), which catalyzes the reversible transformation of glucose-1-phosphate and glucose-6-phosphate. PGM participate in the provision of glucose-1-phosphate required for the synthesis of sugar nucleotide precursors such as ADP-glucose required for glycogen synthesis and UDP-glucose required for LPS core components synthesis [Klein and Raina 2015]. *E. coli* Δ*pgm* mutants show alterations in morphology with reductions in size indicating altered envelope structure [Lu and Kleckner 1994].
phoP (Envelope integrity)**	C	*phoP* encodes PhoP, a member of the two-component regulatory system PhoP/PhoQ involved in sensing divalent cations such as Mg^{2+}. PhoP is involved in a complex network of reactions modulated by small RNAs responsible of controlling lipid A and LPS composition and heterogeneity, as well as LPS translocation to the OM. *E. coli* Δ*pho*P mutants contain LPS structural defects likely triggering the RpoE stress response. PhoP/PhoQ participates in bile resistance in *Salmonella enterica* [Begley et al. 2005, Klein and Raina 2015, Klein et al. 2016].
phoU (Envelope integrity; DNA replication, recombination and/or repair)*	C	*phoU* encodes one of the negative regulators of the *pho* regulon involved in phosphate metabolism mediated by the interaction with the phosphate sensor kinase PhoR of the PhoR/PhoB two-component system. *phoU* forms part of the *pstSCAB-phoU* operon also encompassing *pstA* and *pstC* identified in this group (see below). *E. coli* Δ*phoU* mutants show constitutive PhoB kinase production leading to high expression of the *pho* regulon, including *eptC*, *waaH*, and *ugd* genes involved in LPS core and lipid A modifications suggesting altered envelope integrity. Δ*phoU* mutants also show defects in the mutagenic repair of DNA breaks and severe growth defects [Steed and Wanner 1993, Al Mamun et al. 2012, Gardner et al. 2014, Gibson et al. 2015, Klein and Raina 2015].
pphA (Envelope integrity)*	C	*pphA/prpA* encodes a phosphoprotein phosphatase involved in signalling periplasmic protein misfolding in a pathway that includes the CpxR/CpxA two-component system. *pphA* belongs to the RpoH heat shock regulon [Missiakas and Raina 1997].
prc	IM, P	*prc* encodes a periplasmic protease involved in the cleavage of a C-terminal peptide from the precursor form of penicillin-binding protein 3

(Osmoprotection)		(PBP3). *E. coli* Δ*prc* cells show altered morphologies in high-salinity medium. May be involved in protection from thermal and osmotic stresses [Kerr et al. 2014].
prfB	C	peptide chain release factor RF2
proA	C	*proA* encodes glutamate-5-semialdehyde dehydrogenase, involved in proline biosynthesis. Loss of proline production may affect *E. coli* osmotolerance [Smith 1985]
proQ (Osmoprotection)	C	*proQ* encodes a protein with RNA chaperone activity involved in the post-transcriptional control of ProP, an osmolyte:H^+ symporter of the inner membrane. *proQ* deficiency decreases the level of the osmoregulatory glycine-betaine transporter ProP, reduces growth and affects cell morphology under high medium osmolarity [Kerr et al. 2014].
proX (Osmoprotection)	P	*proX* encodes a glycine-betaine periplasmic binding protein component of the ABC-type transporter ProU, responsible of translocating a wide range of compatible solutes contributing to the regulation of cell volume under fluctuations of medium osmolarity.
prpE	C	*prpE* encodes a propionate-CoA ligase. cAMP/CRP regulon member.
pstA	IM	*pstA* encodes the phosphate permease subunit of an ABC-type transporter. Forms part of the *pstSCAB-phoU* operon of the *pho* regulon, with *pstC* and *phoU* also identified in this screening.
pstC	IM	*pstC* encodes a phosphate permease subunit of the ABC transporter type. Forms part of the *pstSCAB-phoU* operon also encompassing *pstA* and *phoU* identified in this group.
recB (DNA replication, recombination and/or repair)	C	*recB* encodes a subunit of the RecBCD Exonuclease V subunit, involved in DNA recombination and repair [Al Mamun et al. 2012].
rfaC (Envelope	C, IM	*rfaC/waaC* encodes LPS heptosyltransferase I. It forms part of the *rfaD-waaFCL* operon involved in LPS core biosynthesis. Δ*rfaC* mutants show

integrity)**		envelope defects as indicated by an increased susceptibility to anionic detergents and high temperature (45-47 °C) and constitutive expression of RpoE [Murata et al. 2011; Nicolaes et al. 2014, Klein and Raina 2015].
rfaE<br>(Envelope integrity)**	C	*rfaE/waaE* encodes a heptose 7-P kinase/heptose 1-P adenyltransferase envolved in LPS core synthesis. *E. coli* Δ*rfaE* mutants show envelope defects as indicated by the increased susceptibility to anionic detergents and high temperature (45-47 °C [Murata et al. 2011, Nicolaes et al. 2014, Klein and Raina 2015].
rfaF<br>(Envelope integrity)**	C	*rfaF/waaF* encodes ADP-heptose:LPS heptosyltransferase II, a member of the *rfaD-waaFCL* operon involved in LPS core biosynthesis from which *waaC* was also identified in this screening (see above). *E. coli* Δ*rfaF* mutants show envelope defects as indicated by the increased susceptibility to anionic detergents and high temperatures (45-47 °C), and elevated RpoE levels [Murata et al. 2011; Nicolaes et al. 2014, Klein and Raina 2015].
rfaH<br>(Envelope integrity)**	C	*rfaH* encodes RfaH, a transcription antitermination factor which positively controls transcription of LPS biosynthesis genes of the *waaQGPSBOJYZU* operon. *E. coli* Δ*rfaH* mutants show envelope defects as indicated by the increased susceptibility to anionic detergents [Nicolaes et al. 2014, Klein and Raina 2015].
rfaP<br>(Envelope integrity)**	IM	*rfaP/waaP* encodes lipopolysaccharide core heptose (I) kinase. *rfaP* is a member of the *waaQGPSBOJYZU* operon positively controlled by RfaH, also identified in this screening. *E. coli* Δ*rfaP* mutants show envelope defects as indicated by increased susceptibility to anionic detergents Nicolaes et al. 2014, Klein and Raina 2015].
rpsT	C	30S ribosomal subunit protein S20
rseA<br>(Envelope integrity)**	IM, C	*rseA* encodes an anti-RpoE sigma factor that sequesters RpoE in the inner membrane thus preventing its interaction with the core RNA polymerase to activate transcription of genes required to respond to envelope stress. *E. coli* Δ*rseA* mutants thus contain elevated RpoE levels leading to an unregulated envelope stress response that results in global alterations in LPS composition and envelope integrity, as indicated by an increased susceptibility to anionic detergents and high (45-47 °C) temperature [Murata et al. 2011, Klein and Raina 2015, Nicolaes et al. 2014].

ruvC<br><br>(DNA replication, recombination and/or repair, Envelope integrity)*	C	*ruvC* encodes a component of the RuvABC resolvasome which binds to and cleaves Holliday junctions generated during repair of DNA breaks. This also results in a number of chromosome dimers that are resolved by XerC/D recombinases (also identified here, see below). Interruption of this process leads to impairements in chromosome segregation and cell division. *E. coli* Δ*ruvC* mutants show increased susceptibility to high (45-47 °C) temperature suggesting envelope alterations [Al Mamun et al. 2012, Murata et al. 2011, Buljubašić et al. 2013].
slyB<br><br>(Envelope integrity)*	OM	*slyB* encodes an OM lipoprotein structured in homo-oligomeric complexes of unknown function [Maddalo et al. 2011]. Removal of this protein from the OM may result in envelope defects and an RpoE-mediated response.
surA<br><br>(Envelope integrity)**	P	*surA* encodes a peptidyl-prolyl *cis-trans* isomerase necessary for the proper folding/assembly of OM proteins including OmpA, OmpC, and the essential LPS assembly factor LptD. Δ*surA* mutants show increased permeability to anionic detergents and envelope defects including LPS alterations and deficient OM protein assembly leading to the induction of the RpoE stress response [Ruiz and Silhavy 2005, Rigel and Silhavy 2012, Nicolaes et al. 2014].
tatA<br><br>(Envelope integrity)**	IM	The *tatA* gene product is a component of the TatABCE (twin-arginine translocation) protein translocation complex mediating export of folded and ligand-bound proteins. *E. coli* mutants lacking a functional Tat system show outer membrane defects and cell division impairements [Stanley et al. 2001, Bernhardt and de Boer 2003, Ize et al. 2003, Peters et al. 2011, Black et al. 2013].
tatB<br><br>(Envelope integrity)**	IM	The *tatB* gene product is another component of the TatABCE protein translocation system (see above). *E. coli* Δ*tatB* mutants show envelope integrity defects and increased susceptibility to SDS (Ize et al. 2003, Nicolaes et al. 2014).
tatC<br><br>(Envelope integrity)**	IM	The *tatC* gene product is another component of the TatABCE protein translocation system (see above). *E. coli* Δ*tatC* mutants show envelope integrity defects and increased susceptibility to SDS (Ize et al. 2003, Nicolaes et al. 2014). *tatC* was identified in a screening of genes involved in DNA repair by homologous recombination [Al Mamun et al. 2012].

tolB<br><br>(Envelope integrity)**	P	Tol B is a periplasmic protein member of the Tol-Pal complex localizing at the divisome and required for OM invagination during septation. *E. coli* Δ*tolB* mutants show envelope integrity defects and increased susceptibility to SDS [Nicolaes et al. 2014; Typas et al. 2012]. Other proteins also identified in this screening which are recruited or interact with the divisome are DedD, MrcB, (PBP1B), and YcfM (LpoB).
tolQ<br><br>(Envelope integrity)**	IM	TolQ is one of three inner membrane proteins, along with TolA and TolR, composing the Tol-Pal complex. *E. coli* Δ*tolQ* mutant shows envelope integrity defects as inferred from their increased susceptibility to SDS and high temperature (45-47 °C) [Murata et al. 2011; Nicolaes et al. 2014]. TolQ participates in bile resistance in *Salmonella enterica* [Begley et al. 2005].
tolR<br><br>(Envelope integrity)**	IM	TolR is one of three inner membrane proteins, along with TolA and TolQ, forming the Tol-Pal complex. *E. coli* Δ*tolR* mutant shows envelope integrity defects as inferred from their increased susceptibility to SDS and high temperature (45-47 °C) [Murata et al. 2011; Nicolaes et al. 2014]. TolR participates in bile resistance in *Salmonella enterica* [Begley et al. 2005]. See also above.
xerC<br><br>(DNA replication, recombination and/or repair; Envelope integrity)*	C	*xerC* encodes a component of the XerCD recombinase involved in the resolution of chromosome dimers recruited at the septal ring by the DNA translocase FtsK to coordinate chromosome segregation with cell division. *xerC* mutants form filaments unable to partition correctly and show increased susceptibility to high temperature (45-47 °C) suggesting envelope alterations [Blakely et al. 1991, Murata et al. 2011, Typas et al. 2012; Weiss 2004].
xerD<br><br>(DNA replication, Envelope integrity)*	C	*xerD* encodes the second component of the XerCD recombinase. For details see above.
ybgC<br><br>(Envelope	IM, C	*ybgC* encodes an ACP-binding protein with thioesterase activity which forms a complex in the IM with the phospholipid synthetic enzymes PssA (phosphatidylserine synthetase) and PlsB (glycerol-3-phosphate acyltransferase) catalyzing the first committed step in phospholipid

integrity)*		biosynthesis leading to phosphatidylglycerol. *ybgC* is the first gene of the *ybgC-tolQRA* operon, and its deletion may have polar effects on the expression of the downstream *tol* genes also identified in this screening (see above) [Parsons and Rock 2013].
ycfM (Envelope integrity)**	OM	*ycfM* encodes LpoB, an outer membrane lipoprotein that activates the bifunctional PBP1B (also identified in this screening, see above) involved in cell division. LpoB is located at the divisome complex during the late stages of divisome assembly required for OM invagination during septation. Also identified in this screening are EnvC, DedD, and Tol-Pal complex proteins which are also recruited or interact with the divisome [Typas et al. 2012].
yciM (Envelope integrity)**	IM	*yciM* encodes an inner membrane protein required for the down-regulation of LPS synthesis by modulating the levels of the lipid A synthesis enzyme LpxC in an FtsH-dependent manner. *E. coli* Δ*yciM* mutants show envelope integrity defects as indicated by their increased susceptibility to SDS and high temperature (45-47 °C). The *yciM* and *envC* genes (also uncovered in this screen, see above) genetically interact (Murata et al. 2011, Nicolaes et al. 2014).
yciS (Envelope integrity)*	IM	*yciS/lapA* encodes LapA, the LPS assembly protein A, and forms with *yciM/lapB* the *yciSM* operon. LapB coordinates assembly of proteins involved in LPS synthesis at the plasma membrane and regulates turnover of LpxC, thereby ensuring balanced LPS/phospholipid biosynthesis. *E. coli yciM* conditional mutants show increased susceptibility to anionic detergents and an elevated RpoE-mediated response indicating envelope defects [Nicolaes et al. 2014; Klein and Raina 2015]. Deletion of *yciS* may thus have polar effects on downstream *yciM.*
ydaS (DNA replication, recombinatio n and/or repair)	?	*ydsA* encodes a putative Cro-like repressor. *E. coli* Δ*ydaS* mutants are more susceptible to base alkylating agents, suggesting roles in DNA repair [Rooney et al. 2009].
ydgI (Purine/thym idylate	IM	Predicted basic amino acid antiporter of the AraD family. *ydgI* forms an operon with the downstream *folM* gene encoding an alternative dihydrofolate reductase involved in C1 metabolism and capable of replacing the main dihydrofolate reductase FolA in its absence. [Levin et

biosynthesis)		al. 2004]. The *ydgI* deletion may have polar effects on the downstream *folM* gene.
yfgJ	C	Required for swarming phenotype, function unknown
ygeG	C	*ygeG*, located in a cryptic type III secretion system (T3SS) of *E. coli* K-12, encodes a SicA-like chaperone of unknown function [Ren et al. 2004]
ygfA (purine biosynthesis)	IM	*ygfA* encodes 5-formyltetrahydrofolate cycloligase (YgfA) required for the removal of toxic 5-formyltetrahydrofolate (5-CHO-THF), a side product of the synthesis of 5,10-methylene-tetrahydrofolate by serine hydroxymethyltransferase. 5-CHO-THF is a potent inhibitor of various folate-dependent enzymes of the C1 metabolism network, including enzymes of both the thymidylate and purine biosynthetic pathways. [Jensen et al. 2008, Jeanguenin et al. 2010].
yhcB	IM	Uncharacterized membrane-anchored protein
yhdP	IM	Conserved membrane protein, predicted transporter.
yigZ	C	Function unknown, UPF0029 family, IMPACT family; distantly related to elongation factors
yiiS (DNA replication, nutrient starvation)	C	*yiiS* encodes an UPF0381 family protein, function unknown. The *yiiS* gene forms part of the *yiiS-uspD* operon under control of an RpoE-dependent promoter. The *uspD* gene encodes UspD, the universal stress protein D involved in defense against DNA damage, nutrient starvation, and other stress situations [Gustavsson et al. 2002]. The *yiiS* deletion may thus have polar effects on the transcription of the downstream *uspD* gene.

References

Al Mamun, A.A., *et al*. Identity and function of a large gene network underlying mutagenic repair of DNA breaks. *Science*. **338**, 1344-48 (2012).

Bay, D.C. & Turner, R.J. Small multidrug resistance protein EmrE reduces host pH and osmotic tolerance to metabolic quaternary cation osmoprotectants. *J. Bacteriol.* **194**, 5941-48 (2012).

Batchelor, E., Walthers, D., Kenney, L.J. & Goulian, M. The *Escherichia coli* CpxA-CpxR envelope stress response system regulates expression of the porins *ompF* and *ompC*. *J Bacteriol.* **187**, 5723-31 (2005).

Begley, M., Gahan, C.G. & Hill, C. The interaction between bacteria and bile. *FEMS Microbiol. Rev.* **29**, 625-51 (2005).

Bernhardt, T.G. & de Boer, P.A. The *Escherichia coli* amidase AmiC is a periplasmic septal ring component exported via the twin-arginine transport pathway. *Mol Microbiol.* **48**, 1171-82 (2003).

Black, S.L., Dawson, A., Ward, F.B. & Allen, R.J. Genes required for growth at high hydrostatic pressure in *Escherichia coli* K-12 identified by genome-wide screening. *Plos One*. **8**(9), e73995, https://doi:10.1371/journal.pone.0073995 (2013).

Blakely, G., Colloms, S., May, G., Burke, M. & Sherratt, D. *Escherichia coli* XerC recombinase is required for chromosomal segregation at cell division. *New Biol.* **3**, 789-98 (1991).

Buljubašić, M., Zahradka, D. & Zahradka, K. RecQ helicase acts before RuvABC, RecG and XerC proteins during recombination in recBCD sbcBC mutants of *Escherichia coli*. *Res Microbiol*. **164**, 987-97 (2013).

Camberg, J.L., Hoskins, J.R. & Wickner, S. The interplay of ClpXP with the cell division machinery in *Escherichia coli*. *J Bacteriol*. **193**, 1911-18 (2011).

Clegg, S.J., Jia, W. & Cole, J.A. Role of the *Escherichia coli* nitrate transport protein, NarU, in survival during severe nutrient starvation and slow growth. *Microbiology* **152**, 2091-2100 (2006).

Gardner, S.G., Johns, K.D., Tanner, R. & McCleary, W.R. The PhoU protein from *Escherichia coli* interacts with PhoR, PstB, and metals to form a phosphate-signaling complex at the membrane. *J Bacteriol*. **196**, 1741-52 (2014).

Ge, X., *et al*. DegP primarily functions as a protease for the biogenesis of β-barrel outer membrane proteins in the Gram-negative bacterium *Escherichia coli*. *FEBS J*. **281**, 1226-40 (2014).

Gibson, J.L., *et al*. Atypical role for PhoU in mutagenic break repair under stress in *Escherichia coli*. *PLoS One*. **10**(5), e0123315, https://doi:10.1371/journal.pone.0123315 (2015).

Goemans, C., Denoncin, K. & Collet, J.F. Folding mechanisms of periplasmic proteins. *Biochim Biophys Acta*. **1843**, 1517-28 (2014).

Gustavsson, N., Diez, A. & Nyström, T. The universal stress protein paralogues of *Escherichia coli* are co-ordinately regulated and co-operate in the defence against DNA damage. *Mol Microbiol*. **43**, 107-17 (2002).

Ize, B., Stanley, N.R., Buchanan, G. & Palmer, T. Role of the *Escherichia coli* Tat pathway in outer membrane integrity. *Mol Microbiol*. **48**, 1183-93 (2003).

Jeanguenin, L., *et al*. Moonlighting glutamate formiminotransferases can functionally replace 5-formyltetrahydrofolate cycloligase. *J Biol Chem*. **285**, 41557-66 (2010).

Jensen, K., Dandanell, G., Hove-Jensen, B. & WillemoËs, M. Nucleotides, nucleosides, and nucleobases. *EcoSal Plus*. **3**(1), http://doi:10.1128/ecosalplus.3.6.2 (2008).

Jiang, S.S., *et al*. (2010) Characterization of UDP-glucose dehydrogenase and UDP-glucose pyrophosphorylase mutants of *Proteus mirabilis*: defectiveness in polymyxin B resistance, swarming, and virulence. *Antimicrob Agents Chemother*. **54**, 2000-9 (2010).

Kerr, C.H., Culham, D.E., Marom, D. & Wood, J.M. Salinity-dependent impacts of ProQ, Prc, and Spr deficiencies on *Escherichia coli* cell structure. *J Bacteriol*. **196**, 1286-96 (2014).

Klein, G. & Raina, S. Regulated control of the assembly and diversity of LPS by noncoding sRNAs. *Biomed Res Int*. **2015**, 153561 (2015).

Klein, G., *et al.* Multiple transcriptional factors regulate transcription of the *rpoE* gene in *Escherichia coli* under different growth conditions and when the lipopolysaccharide biosynthesis is defective. *J. Biol. Chem.* **291**, 22999-23019 (2016).

Kneidinger, B., *et al.* Biosynthesis pathway of ADP-L-glycero-beta-D-manno-heptose in *Escherichia coli. J Bacteriol.* **184**, 363-69 (2002).

Levin, I., Giladi, M., Altman-Price, N., Ortenberg, R. & Mevarech, M. An alternative pathway for reduced folate biosynthesis in bacteria and halophilic archaea. *Mol Microbiol*. **54**, 1307-18 (2004).

Lu, M. & Kleckner, N. Molecular cloning and characterization of the *pgm* gene encoding phosphoglucomutase of *Escherichia coli*. *J Bacteriol*. **176**, 5847-51 (1994).

Maddalo, G., *et al*. Systematic analysis of native membrane protein complexes in *Escherichia coli*. *J Proteome Res*. **10**, 1848-59 (2011).

Missiakas, D. & Raina, S. Signal transduction pathways in response to protein misfolding in the extracytoplasmic compartments of *E. coli*: role of two new phosphoprotein phosphatases PrpA and PrpB. *EMBO J*. **16**, 1670-85 (1997).

Murata, M., *et al.* Molecular strategy for survival at a critical high temperature in *Escherichia coli. PLoS One.* **6**(6), e20063, https://doi:10.1371/journal.pone.0020063 (2011).

Nicolaes,V., *et al*. Insights into the function of YciM, a heat shock membrane protein required to maintain envelope integrity in *Escherichia coli*. *J Bacteriol*. **196**, 300-9 (2014).

Ohara, M., Wu, H.C., Sankaran, K. & Rick, P.D. Identification and characterization of a new lipoprotein, NlpI, in *Escherichia coli* K-12. *J Bacteriol*. **181**, 4318-25 (1999).

Parsons, J.B. & Rock, C.O. Bacterial lipids: metabolism and membrane homeostasis. *Prog Lipid Res*. **52**, 249-76 (2013).

Peters, N.T., Dinh, T. & Bernhardt, T.G. A fail-safe mechanism in the septal ring assembly pathway generated by the sequential recruitment of cell separation amidases and their activators. *J Bacteriol*. **193**, 4973-83 (2011).

Pucciarelli, M.G., Prieto, A.I., Casadesús, J. & García-del Portillo, F. Envelope instability in DNA adenine methylase mutants of *Salmonella enterica*. *Microbiology*. **148**, 1171-82 (2002).

Ren, C.P., *et al*. The *ETT2* gene cluster, encoding a second type III secretion system from *Escherichia coli*, is present in the majority of strains but has undergone widespread mutational attrition. *J Bacteriol*. **186**, 3547-60 (2004).

Rigel, N.W. & Silhavy, T.J. Making a beta-barrel: assembly of outer membrane proteins in Gram-negative bacteria. *Curr. Opin. Microbiol.***15**, 189-193 (2012).

Rooney, J.P., *et al*. Systems based mapping demonstrates that recovery from alkylation damage requires DNA repair, RNA processing, and translation associated networks. *Genomics*. **93**, 42-51 (2009).

Ruiz, N. & Silhavy, T.J. Sensing external stress: watchdogs of the *Escherichia coli* cell envelope. *Curr Opin Microbiol.* **8**, 122-26 (2005).

Smith, L.T. Characterization of a gamma-glutamyl kinase from *Escherichia coli* that confers proline overproduction and osmotic tolerance. *J Bacteriol*. **164**, 1088-93 (1985).

Stanley, N.R., Findlay, K., Berks, B.C. & Palmer, T. *Escherichia coli* strains blocked in Tat-dependent protein export exhibit pleiotropic defects in the cell envelope. *J Bacteriol*. **183**, 139-44 (2001).

Steed, P.M. & Wanner, B.L. Use of the rep technique for allele replacement to construct mutants with deletions of the *pstSCAB-phoU* operon: evidence of a new role for the PhoU protein in the phosphate regulon. *J Bacteriol.* **175**, 6797-6809 (1993).

Templin, M.F., Ursinus, A. & Höltje, J.V. A defect in cell wall recycling triggers autolysis during the stationary growth phase of *Escherichia coli*. *EMBO J.* **8**, 4108-17 (1999).

Typas, A., Banzhaf, M., Gross, C.A. & Vollmer, W. From the regulation of peptidoglycan synthesis to bacterial growth and morphology. *Nat Rev Microbiol.* **10**, 123-36 (2012).

Vertommen, D., *et al*. The disulphide isomerase DsbC cooperates with the oxidase DsbA in a DsbD-independent manner. *Mol Microbiol.* **67**, 336-49 (2008).

Weiss, D.S. Bacterial cell division and the septal ring. *Mol Microbiol.* **54**, 588-597 (2004).

Williams, B., Bhat, N., Chien, P. & Shapiro, L. ClpXP and ClpAP proteolytic activity on divisome substrates is differentially regulated following the *Caulobacter asymmetric* cell division. *Mol Microbiol.* **93**, 853-866 (2014).

Yao, Z., Davis, R.M., Kishony, R., Kahne, D. & Ruiz, N. Regulation of cell size in response to nutrient availability by fatty acid biosynthesis in *Escherichia coli*. *Proc Natl Acad Sci U S A.* **109**, E2561-68 (2012).

Supplementary Figure S1: ADPG consumption by BW25113 *E. coli* cells in liquid KM supplemented with 1,5 mM ADPG. Each plot represents the mean and SEM of one biological replicate with three technical replicates each.

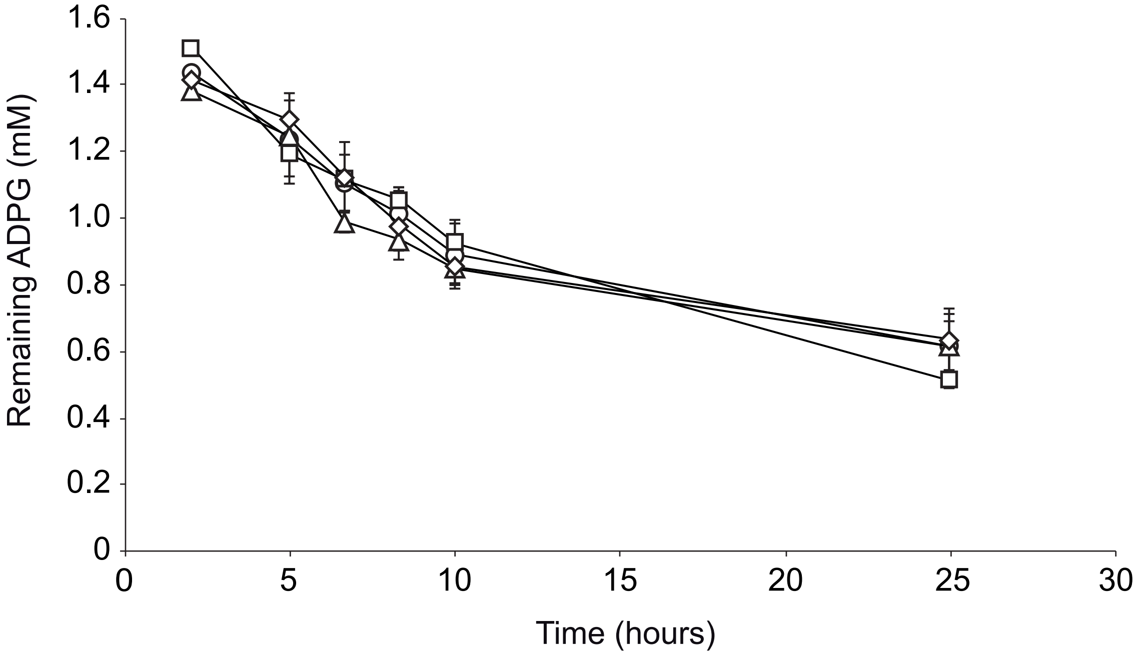

Supplementary Figure S2: Growth of BW25113 *E. coli* cells cultured in liquid M9 medium supplemented with 1,5 mM ADPG, adenosine, AMP or ADP.

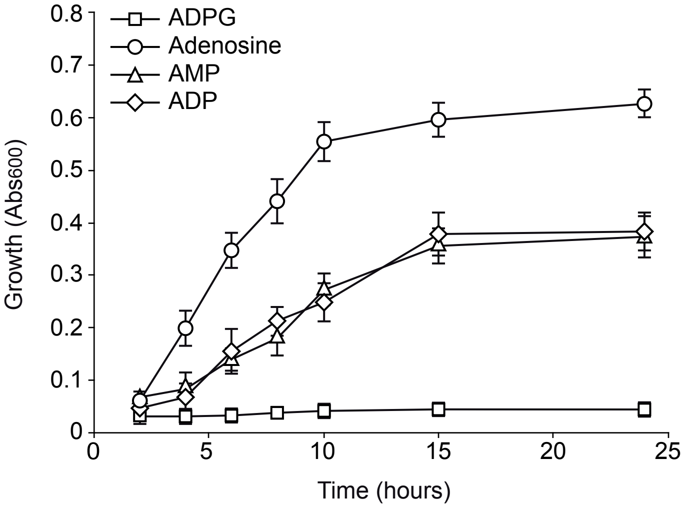

Supplementary Figure S3: Bacterial growth in experiments for which results are shown in Figs 1, 3, 4 and 6-8.

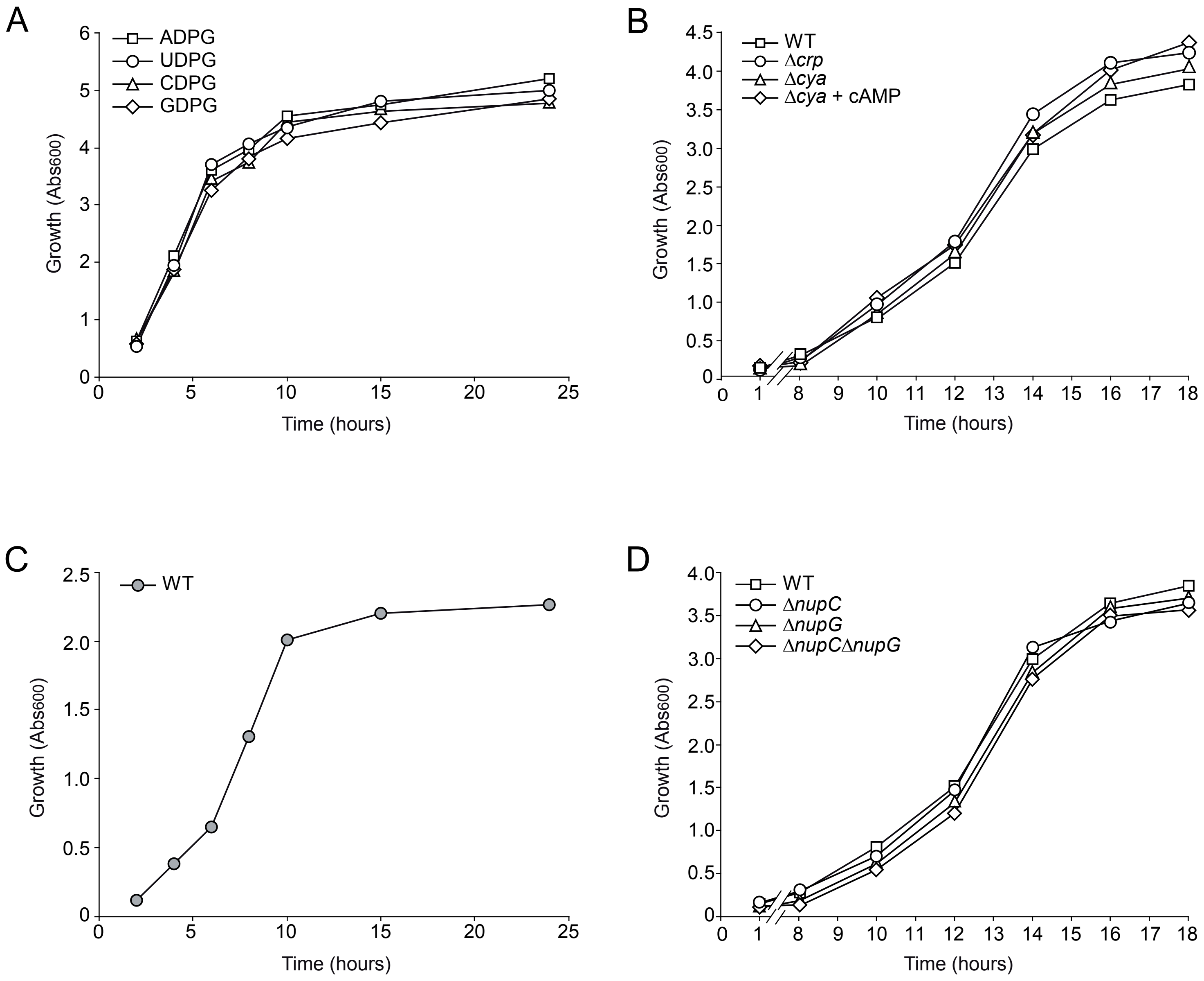

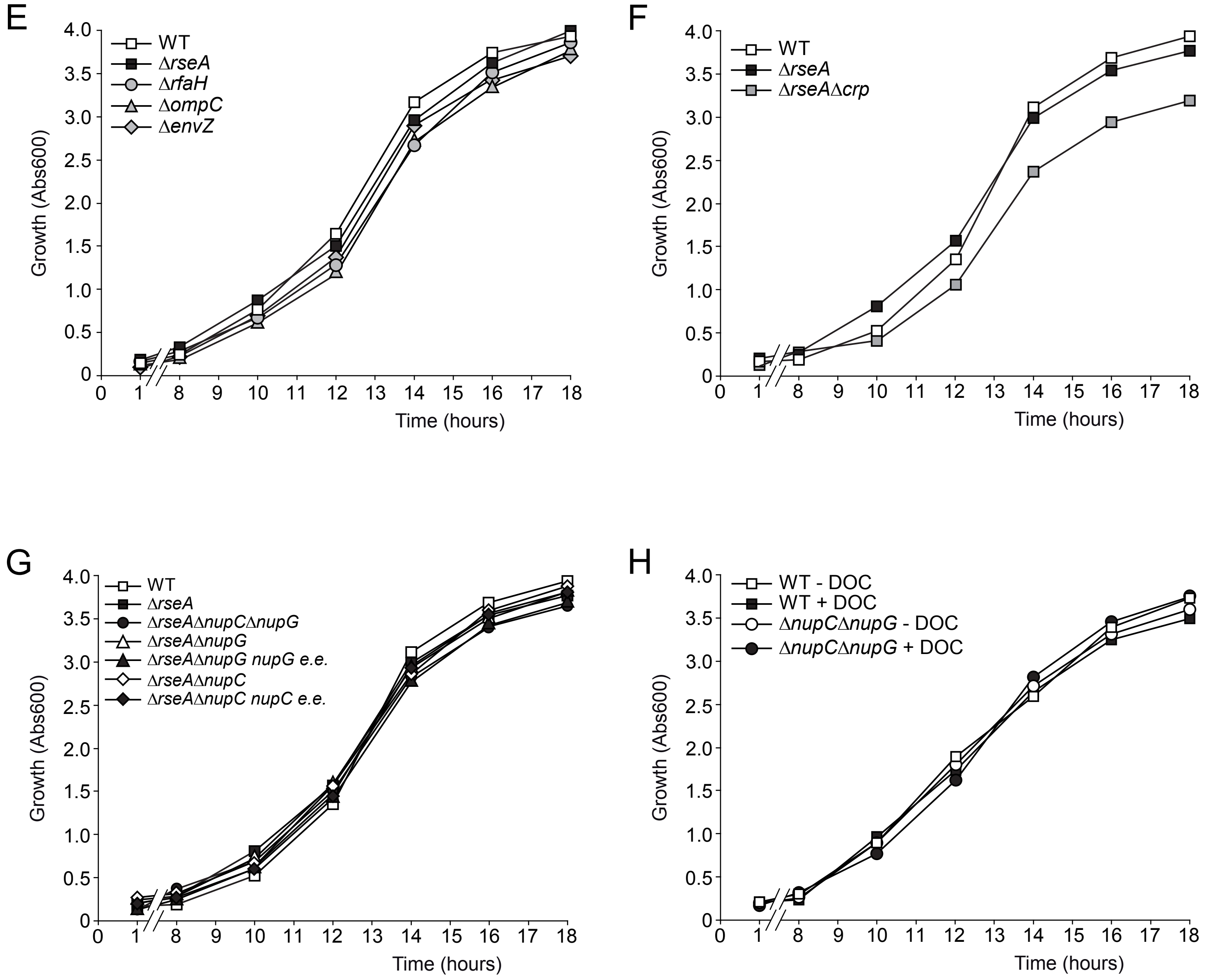
E
WT
ΔrseA
ΔrfaH
ΔompC
ΔenvZ
Growth (Abs600)
Time (hours)
F
WT
ΔrseA
ΔrseAΔcrp
Growth (Abs600)
Time (hours)
G
WT
ΔrseA
ΔrseAΔnupCΔnupG
ΔrseAΔnupG
ΔrseAΔnupG nupG e.e.
ΔrseAΔnupC
ΔrseAΔnupC nupC e.e.
Growth (Abs600)
Time (hours)
H
WT - DOC
WT + DOC
ΔnupCΔnupG - DOC
ΔnupCΔnupG + DOC
Growth (Abs600)
Time (hours)
